# Tetrameric STAT5 regulates the formation of immune niche cells to protect stem cell regenerative repair against mucosal inflammation

**DOI:** 10.1038/s12276-026-01716-0

**Published:** 2026-05-01

**Authors:** Haifeng Li, Xueli Ding, Sabrina Fabec, Ahmed Bakheet, Wen Gao, Mingquan Song, Ruixue Liu, Xiaonan Han

**Affiliations:** 1 Institute of Laboratory Animal Sciences, Peking Union Medical College, Beijing, People’s Republic of China; 2 Division of Hematology and Oncology, and Division of Cancer Biology, Department of Medicine, The MetroHealth System, Case Western Reserve University School of Medicine, Cleveland, OH USA; 3 Department of Gastroenterology, the Affiliated Hospital of Qingdao University, Qingdao, People’s Republic of China; 4 Cancer Genomics and Epigenomics Program, Case Comprehensive Cancer Center, Case Western Reserve University, Cleveland, OH USA

**Keywords:** Ulcerative colitis, Intestinal stem cells, Growth factor signalling, Interleukins

## Abstract

How intestinal stem cells (ISCs) are regulated during inflammation remains largely unexplored, leading to a lack of effective treatments for inflammatory bowel diseases (IBD). ISC-mediated intestinal epithelial regenerative repair can be regulated by intra-epithelial T lymphocytes, whose maturation is controlled by STAT5 dimeric or tetrameric activation. However, the mechanisms by which the T lymphocytes can protect ISCs are unclear. Here we hypothesize that tetrameric STAT5 regulates intra-crypt T cells to act as niche cells for ISC regeneration. Using IBD biospecimens, STAT5-hyperactive, tetramer-deficient mice and organoids, we found IBD–ulcerative colitis exhibited more crypt TCRγδ^+^STAT5^+^ T cells and less ISC pluripotency than healthy patients. Compared with wild-type mice, depleting tetrameric STAT5 in mice significantly increased ISC-mediated intestinal epithelial hyperplasia, TCR gene signatures, crypt TCRγδ^+^ T cells with elevated STAT5 tyrosine phosphorylation (pYSTAT5) and IL-17A levels, amplified both Lgr5^hi^ and Lgr5^low^ ISC proliferation and promoted de novo crypt regeneration with increased TCRγδ^+^ cell influx post irradiation or colitis. By contrast, depleting tetrameric STAT5 in organoids reduced ISC pluripotency and organoid growth post irradiation. Mechanistically, chromatin immunoprecipitation and single-cell RNA sequencing analyses with crypt cells revealed that depleting STAT5 tetramers decreased STAT5-binding on the *Metallothionein 1* (*Mt1*) locus in crypt T cells and increased *Mt1* expression, which leads to T cell migration into crypts and enhanced ISC regeneration. Together, the tetrameric STAT5 suppresses the formation of the crypt T cell niche. Interrupting STAT5 tetramers promotes the expansion of crypt TCRγδ cells, providing a target for promoting ISC regenerative repair during IBD–ulcerative colitis.

## STAT5 tetramers control T cell niche for ISC repair

Inflammatory bowel disease (IBD) presents significant therapeutic challenges due to limited understanding of intestinal stem cell (ISC) regeneration. This study identifies a novel role for TCRγδ T cells in the crypts of patients with IBD, particularly those with ulcerative colitis, highlighting their potential in promoting ISC regeneration. Using genetically engineered mouse models, the authors demonstrate that depleting tetrameric STAT5 enhances the presence of TCRγδ T cells in crypts, which secrete IL-17A to support ISC repair. The study employs a combination of RNA sequencing, immunohistochemistry and organoid cultures to elucidate the mechanisms by which STAT5 tetramer depletion facilitates T cell infiltration and ISC regeneration. These findings suggest that targeting on reducing STAT5 tetramers could enhance mucosal healing in IBD by promoting a supportive immune niche for ISCs.

This summary was initially drafted using artificial intelligence, then revised and fact-checked by the author.

## Introduction

Inflammatory bowel disease (IBD) refers to a group of debilitating conditions that can be life-threatening without timely treatment^1^. However, therapies to promote gastrointestinal healing are lacking due to our limited understanding of potential targets for controlling mucosal damage^2^. Moreover, a lack of mechanistic studies on IBD healing has limited current therapeutics to inflammation suppression, leading to the unavoidable use of systemic steroidal drugs that induce debilitating side effects in patients with IBD with long-term use^2,3^. Therefore, it is imperative to identify reliable targets that control gastrointestinal healing. Intestinal stem cells (ISCs), particularly those expressing leucine-rich-repeat-containing G-protein-coupled receptor 5 (LGR5), give rise to intestinal epithelial cell (IEC) lineages that maintain IEC functions and mediate intestinal healing. Impairment of ISC regeneration is believed to interfere with IEC healing in IBD and may occur even during the absence of visible mucosal lesions^4^. Niche cells are associated with ISCs or surround crypt bases, including adjacent mesenchymal cells^5^, Paneth-like cells^6^ and immune cells^7^, can regulate ISC regenerative responses to intestinal infection and inflammation by juxtacrine (direct) or paracrine (diffuse) ligand–receptor interactions^8^. Notably, several studies suggest that loss of immune niche cells may trigger impaired ISC regeneration, predisposing individuals to IBD^9,10^. However, the role of immune niche cells in ISC regeneration is largely unexplored.

LGR5^+^ ISCs express high levels of major histocompatibility complex (MHC) I and II, which are pivotal for regenerative repair. During this process, ISCs directly interact with TCR on T helper (T_H_) cells. They are rapidly transformed into progenitors at the transit-amplifying (TA) zone, which can give rise to differentiated cell lineages^11–13^. ISC differentiation and function are also regulated by cytokines secreted from mucosal T_H_1, T_H_2 and T_H_17 cells. For example, IL-10 can promote ISC self-renewal, whereas IL-17A halts ISC self-renewal, inducing ISC differentiation into secretory lineages^11^. Importantly, IL-17A can directly protect ISC regeneration against experimental colitis by binding IL-17RA on Lgr5 ISCs^14^. Intriguingly, allogeneic T cells can be recruited to the crypt compartment after transplantation, resulting in the loss of ISCs, and T cell-derived IFN-γ can directly target ISCs, leading to their depletion^15,16^. Moreover, integrin αEβ7-expressing T cells can regulate ISC fate through interaction between integrin αEβ7 and E-cadherin in ISCs^17^. However, the mechanisms and consequences of T cell infiltration into the crypt bases are unknown; the effects of IL-17A dosage on Lgr5 ISC behaviors have not been thoroughly studied, and whether these cells can be targeted to protect ISCs remains unclear.

The transcription factors STAT5A/B act as signaling nodes downstream of various mucosal cytokines, growth factors, and hormone–JAK pathways, which induce STAT5 tyrosine phosphorylation (pYSTAT5), leading to dimerization and activation^18^. STAT5A is required for ISC self-renewal and IEC barrier function, and STAT5B activation can maintain intestinal T cell function^19–21^. The genetically engineered mouse model in which the *Stat5* genes (both *Stat5a* and *Stat5b*) are flanked by loxP DNA sequences, allowing conditional deletion of the dimerized STAT5A and STAT5B proteins in specific cells or tissues when crossed with mice expressing Cre recombinase^22^. Using *Stat5* floxed mice, loss of STAT5 dimerization was found to inhibit intestinal T cell function and ISC regeneration as an intrinsic factor, exacerbating mucosal inflammation^19,23,24^. By mutating S711 on STAT5A (S711F), which results in the constitutively activated STAT5 (cS5), our group generated floxed mice with cS5 knock-in. Activating cS5 in Lgr5 ISCs can enhance their resistance to radiation injury^19^.

Critically, STAT5 is a modular transcription factor composed of an N-terminal domain that enables tetramerization^21,25,26^. STAT5 tetramers can bind motifs distinct from those bound by STAT5 dimers to induce or repress gene expression, such as *c-Myc*, *Bcl-2* and *Cyclin D2*^27,28^. In particular, whereas cytokine-activated STAT5 dimers bind to high-affinity STAT5 TTCn_2~5_GAA γ-interferon-activated sequence (GAS) and GAS-like motifs^29^, N-terminal-domain-mediated STAT5 tetramers bind to low-affinity tandem GAS-like motifs with a spacing distribution of 2–17 bp (refs. ^27,28^). Consensus tetramer motifs are found in genes encoding a number of cytokines, including IL-2, IL-7, IL-15, IL-17A, IL-22, IL-23 and IFN-γ^28,30–33^. I28, F81 and L82 at the N-domain of STAT5A and STAT5 B are required for STAT5 tetramer but not dimer formation^34^. Mutation of these residues alone or in combination does not affect the binding of STAT5 dimers, but it abrogates STAT5 tetrameric binding. Based on this rationale, *Stat5a*–*Stat5b* N-domain double knock-in (DKI) mice were generated, in which normal STAT5 dimers function, but the formation of more stable STAT5 tetramers is blocked^28^. Notably, unlike STAT5 dimer-deficient mice, STAT5 tetramer-deficient mice have fewer CD8^+^ T cells and attenuated mucosal T regulatory (T_reg_) cells (CD4^+^Foxp3^+^) relative to littermate controls^27,28^. T_reg_ cells can also directly suppress intestinal TCRγδ T cells or IL-17 production by upregulating IL-10, suggesting that STAT5 tetramer might function as a TCRγδ suppressor by reducing IL-17 production^35,36^. Moreover, tetrameric STAT5 interacts with the H3K27 methylase EZH2 to form H3K27-trimethylated repressive chromatin^37^ and may directly regulate T cell receptor gamma common (*TCRγ*) gene chromatin accessibility and rearrangement^38^. These findings suggest STAT5 tetramers may function to suppress intestinal TCRγδ T cells and control their function.

By using dimeric or tetrameric STAT5-deficient mice, activated STAT5 transgenic mice, organoids, intestinal and colonic ISC injury models and genomic analysis of patients with IBD, our Article, for the first time, demonstrates that crypt T cells are TCRγδ T cells that play a protective role in ISC regeneration by secreting IL-17A. The crypt TCRγδ T cells can be regulated by STAT5 dimerization activation at the expense of STAT5 tetramers that generate a suppressive chromatin. The crypt TCRγδ T cells were increased in patients with IBD–ulcerative colitis (UC) and may be regulated by STAT5 activation, implicating their role in crypt repair during mucosal inflammation.

## Materials and methods

### Materials

All chemicals and antibodies were purchased from Sigma-Aldrich unless otherwise noted. Details are listed in the Supplementary Information.

### Biopsies and surgical specimens from patients with IBD

Colonic biopsy specimens and blood samples from adult patients^39^, including 21 with CD, 24 with UC and 10 healthy controls, were obtained from the Department of Gastroenterology at the Affiliated Hospital of Qingdao University, Qingdao, Shandong, People’s Republic of China. The details of patient samples are listed in Supplementary Table 1. Paraffin-embedded surgical specimens were from the division of pathology, MetroHealth Medical Center (MHMC), Cleveland, USA, and the division of pathology, University of Cincinnati Medical Center, Cincinnati, USA.

Before processing for hematoxylin and eosin (HE), histology, RNA extraction and patient enrollment, institutional review board (IRB) or de-identified IRB protocols for using samples of patients with IBD were approved, respectively, by IRB protocols at MHMC/CWRU, Cleveland, OH, USA (XH, IRB21-00237), the ethics committee of Affiliated Hospital of Qingdao University, Qingdao, People’s Republic of China (QYFY-WZLL-28569 and 29546) and de-identified Cincinnati Children’s Hospital Medical Center (CCHMC), Cincinnati, OH, USA (X.H., IRB2009-1680). All research was performed in accordance with relevant guidelines/regulations using human samples.

### Animal resources and maintenance

The animal study protocols were approved by the Institutional Animal Care and Use Committee (IACUC) at MHMC and CWRU, Cleveland, OH, USA (IACUC 2020-0081) and at ILAS, Beijing, Pople’s Republic of China (LHF21001)^39^. All mice were maintained in specific pathogen-free conditions in the MHMC/CWRU and ILAS Animal Care Facility. All experiments were performed in accordance with the relevant guidelines and regulations for live vertebrates. Inducible *Stat5* depletion (*Stat5*^−/−^) and constitutively active *Stat5* mice (cS5) and *Stat5a*–*Stat5b* DKI N-domain mutant mice (DKI, hereafter called STAT5 tetramer-deficient mice) were generated as previously reported^19,28^. *Rosa26-cre*ERT2 (*RsCre*ER) mice were purchased from Jackson Laboratory (strain no. 008463). Detailed procedures for crossing and genotyping *RsCre*ER;*stat5*^*f/f*^ or *cS5*^*f/f*^, and *Lgr5Cre*ER;DKI mice to inducibly deplete *Stat5* or activate *cS5*, are shown in Supplementary Fig. 1a. Detailed procedures for mouse crossing and genotyping, as well as primer sequences, are described in the Supplementary Fig. 2a and Supplementary Table 2.

### Radiation-induced injury models

The Rs26CerER, cS5 and DKI mice were irradiated with 13 Gy. In brief, DKI and wild-type (WT) control mice were exposed to 13 Gy whole-body γ-radiation at the ILAS or the CWRU Comprehensive Cancer Center Core19. The intestinal tissues were inspected for gross and histological abnormalities. The detailed procedure is described in the Supplementary Information.

### Animal model of colitis

In brief, DKI and WT littermate control mice were orally given 2.5% dextran sulphate sodium (DSS) in water 7 days following with 5-day water recovery, specific for inflammation-induced colonic ISC repair, or three cycles of 5-day DSS following 16-day water recovery, specific for apoptosis-induced colonic ISC regeneration^20^. The detailed method is described in Supplementary Fig. 5b.

### IH, IF and qPCR

Anti-Ki67, lysozyme, apoptosis inhibitor 5 (API5), EdU, CD3, TCRγδ, STAT5 and pYSTAT5 antibodies listed in Supplementary Table 3 were used to detect crypt ISC proliferation, Paneth cell hyperplasia, T cell and STAT5 activation. The expression levels of cytokines (*TNFα*, *IFNγ*, *IL-4*, *6*, *7*, *10*, *12*, *15*, *17*, *22*, *23* and *27*), ISC markers (*Lgr5*, *Ascl*, *Olfm4* and *Lyz1*) and metallothioneins (*Mt1* and *Mt2*) were measured with quantitative PCR (qPCR), the primers of which are listed in Supplementary Table 4. Detailed methods are described in the Supplementary Information.

### FACS analysis

Peripheral blood mononuclear cells (PBMCs) were collected from patients with IBD–UC. Mouse mucosa and crypts of the small intestine (SI) or colon were dissociated into single cells, and the cells were gated into IEC and crypt lymphocyte compartments. Lgr5 fluorescence-activated cell sorting (FACS) analysis^40^ was performed using isolated SI epithelia from *Lgr5Cre*ER and *Lgr5Cre*ER;DKI mice after intraperitoneal administration of EdU at 50 mg/kg. Intra-IEC, lamina propria (LP) or crypt T cells were stained with anti-mouse CD3, CD4, CD8 and TCRγδ antibodies. Human PBMCs were stained with anti-human CD3, CD4, CD8, CD45, TCRαβ or TCRγδ antibodies (Supplementary Figs. 1a and 2b). The antibodies are listed in Supplementary Table 3. A detailed method is described in the Supplementary Information.

### Organoid’s culture, IR treatment, EdU incorporation and medium transfer culture

The intact crypts were dissociated from the SI and the colon. The enteroids or colonoids were cultured^41^. In brief, the organoids were in vitro differentiated from day 1 to day 14, then exposed to 2 Gy IR. After EdU incorporation, the organoids were fixed, or total RNA was extracted with TRIzol. The live organoids or the sections from OCT-embedded organoids were video-recorded or imaged. Lgr5 ISC self-renewal or proliferation, organoid multiplicity and size of spheres were determined. Lgr5 organoid or organoid-forming capacity in the presence or absence of IR or IL-17A treatment was determined by counting final organoid survival and initial-grown organoids (≥200) to calculate the percentage of regenerated organoids. The detailed culture procedure is described in the Supplementary Information.

### RNA-seq analysis

Three healthy controls and seven IBD (UC), three DKI and three littermate control mice at basal conditions, and six DKI and five control mice post IR, were killed. The SIs were isolated and frozen with liquid nitrogen. SI crypts were dissociated from three DKI and three control mice to differentiate the enteroids. Total RNA from either frozen SI tissues or enteroids was extracted for RNA sequencing (RNA-seq)^40^. A detailed method is described in the Supplementary Information and Supplementary Table 5. Bioproject numbers are PRJNA1200789 for human colonic tissues; PRJNA1447506, PRJNA845965 and PRJNA705806 for mouse tissues; and PRJNA721570 for organoids.

### ChIP sequencing

The SI crypts from DKI and littermate control mice were dissociated and used to perform chromatin immunoprecipitation (ChIP) using the simple-ChIP kit according to the manufacturer’s protocol (Cell Signaling Technology and New England Biolabs). The ChIP antibodies are listed in Supplementary Table 3. The detailed method is described in the Supplementary Information. Bioproject numbers are PRJNA1197577 and PRJNA748855.

### scRNA-seq

The mice were killed. The SI were inverted, and mucosal cells were stripped and dissociated for single-cell RNA-seq (scRNA-seq)^11,42^. Cell annotation makers are listed in Supplementary Table 5. A detailed method is described in the Supplementary Information. The bioproject number is PRJNA1180729.

### Transcript profiling

The bioproject numbers for transcript profiling include: PRJNA1200789, PRJNA1447506, PRJNA845965, PRJNA705806 and PRJNA721570 for RNA-seq; PRJNA1197577 and PRJNA748855 for ChIP sequencing; and PRJNA1180729 for scRNA-seq.

### Statistical analysis

All data presented in the organoid culture are representative of at least three repeated experiments. The total number of vertebrate animals used in the experiments exceeds five, with a mixed gender unless otherwise stated. All data are presented as mean values with standard error of the mean (s.e.m.) were used for independent two-tailed Student’s *t*-tests or one-way analysis of variance (ANOVA). All data compilation was done using the statistics software GraphPad Prism (7.0). *P* values of 0.05 or less were considered significant when using *t*-tests and analysis of variance.

## Results

### IBD–UC increased colonic crypt TCRγδ^+^STAT5^+^ T cells

**Fig. 1:**
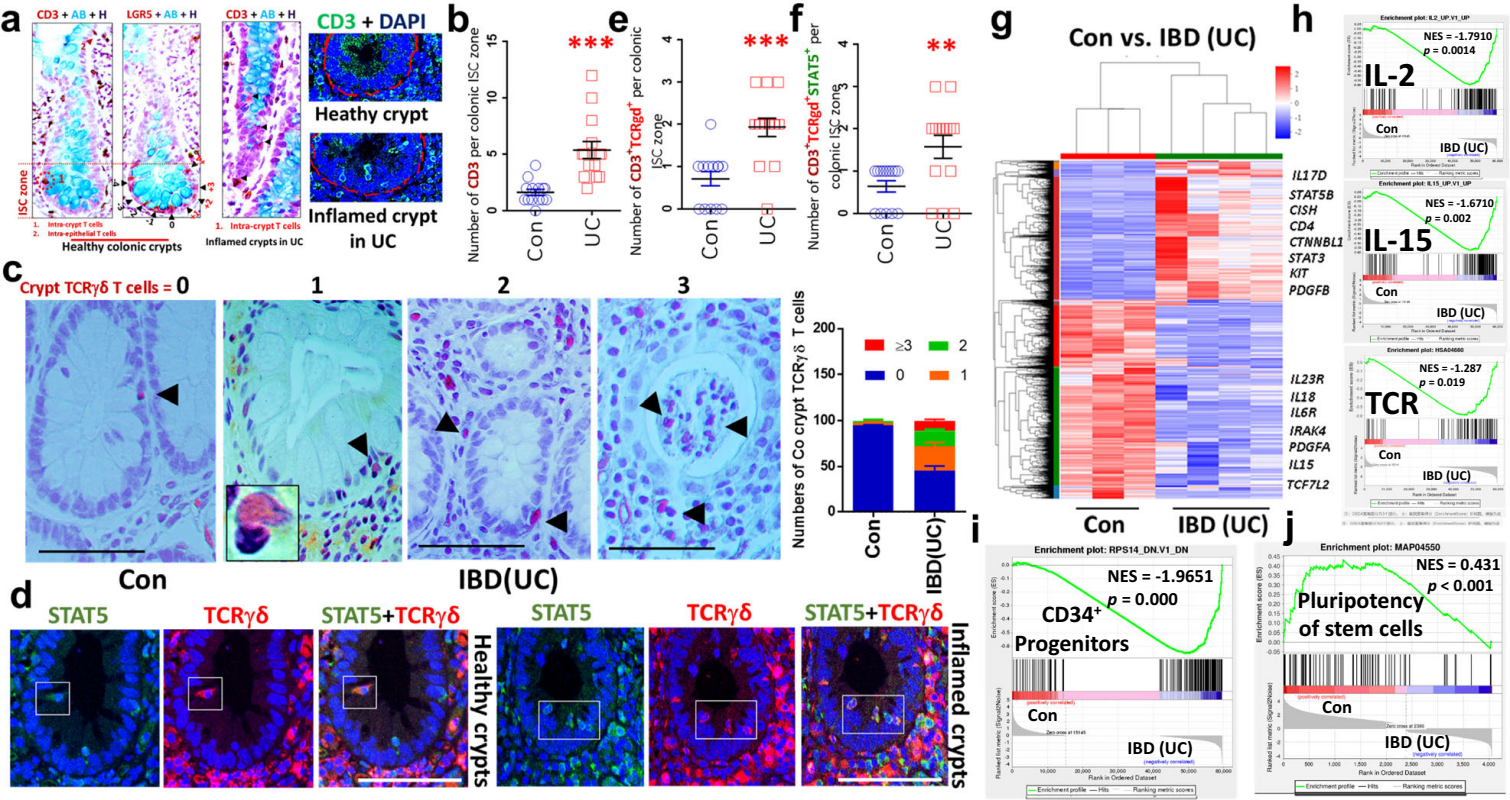
IBD–UC shows increased colonic crypt TCRγδ T cells and TCR gene signatures, while pluripotency gene signatures of stem cells decrease. **a** Paraffin or OCT-embedded slides of crypts from *n* = 10 healthy controls (Con) and *n* = 24 patients with IBD–UC stained with anti-CD3 and anti-LGR5 IH antibodies or anti-CD3 IF antibodies. CD3^+^ T cells were observed in the ISC zone at the bases of colonic crypts in both Con and UC, as indicated by LGR5 staining. AB, Alcian blue; H, hematoxylin. The arrows indicate CD3^+^ T cells or LGR5^+^ ISC. CD3^+^ T cells (green) crypts are represented as open circles. **b** Crypt CD3^+^ T cells were counted in 50–100 well-oriented colonic crypts. Results are expressed as the mean ± s.e.m. ***P < 0.001 versus Con. The arrow indicates crypt TCRγδ T cells. **c** Colon (Co) sections from 10 Con and 24 patients with UC were immunostained with anti-TCRγδ antibodies. TCRγδ^+^ crypt T cells in either healthy or inflamed crypts were quantified as ‘0’, no TCRγδ^+^ T cells, ‘1’, one TCRγδ^+^ T cell, ‘2’, two TCRγδ^+^ T cells, and ‘≥3’, more than three TCRγδ^+^ T cells. The results are expressed as mean ± s.e.m. **d** Double IF staining with anti-TCRγδ (red) and anti-STAT5 (green) antibodies to show STAT5 expression in crypt TCRγδ cells in UC and Con; this image represents *n* = 10 Cons and *n* = 24 UC. **e**, **f** Crypt CD3^+^TCRγδ^+^ T cells (**e**) and TCRγδ^+^STAT5^+^ T cells (**f**) were counted in 50–100 well-orientated colonic crypts to determine the number of positive T cells per crypt ISC stem cell zone. Results are expressed as the mean ± s.e.m., **P < 0.01, ***P < 0.001 versus Con. Colonic tissues were collected from *n* = 3 Cons and *n* = 7 UCs with increased frequency of blood CD3^+^TCRγδ^+^ T cells and crypt TCRγδ^+^ cells. RNA was extracted and subjected to RNA-seq analysis. **g** A heat map shows that compared with Cons, 1644 genes are upregulated and 2,406 genes are downregulated in UC with a fold change >2.0 and *P* < 0.05. **h**–**j** GSEA analysis shows an elevation in the IL-2, IL-15 and TCR pathways (**h**) as well as the CD34^+^ progenitor pathway (**i**) and downregulation of the pluripotency of stem cells pathway in UC versus Con (**j**).

Using FACS analysis, we examined PBMCs from *n* = 24 UC, *n* = 21 Crohn’s disease and *n* = 10 healthy controls (Supplementary Table 1) and found significantly increased frequencies of CD3^+^TCRγδ^+^ T cells in PBMCs from UC but not patients with Crohn’s disease compared with control samples (Supplementary Fig. 1a,b and data not shown). Interestingly, CD3^+^CD4^−^CD8^−^TCRγδ^+^ T cells from these patients with UC show significant activation of pYSTAT5, as indicated by markedly increased frequencies of pYSTAT5^+^CD3^+^TCRγδ^+^ T cells (Supplementary Fig. 1c,d). Consistent with these data, HE and immunohistochemistry (IH) assays with healthy control colonic specimens revealed few intra-IEC lymphocytes, crypt CD3^+^ or TCRγδ^+^ T cells in the healthy crypt compartment, with a significantly elevated number of intra-IEC lymphocytes, CD3^+^ or TCRγδ^+^ T cells in the hyperproliferative, inflamed or cryptitis crypts of patients with UC (Supplementary Fig. 1e and Fig. 1a–c), a finding compatible with a positive role for crypt TCRγδ^+^ cells in ISC regeneration. Moreover, Immunofluorescence (IF) assays showed that part of these infiltrating crypt CD3^+^ T cells in patients with UC are TCRγδ^+^ cells that colocalize with STAT5 staining (Supplementary Fig. 1e and Fig. 1d–f). These data suggest that STAT5 may play a role in recruiting TCRγδ^+^ T cells into the ISC zone or into the crypt base. Importantly, we did not find a significant difference in the frequency of crypt CD3^+^ or TCRγδ^+^ T cells between patients with Crohn’s disease and controls (Supplementary Fig. 1e,f), suggesting that the population of crypt TCRγδ T cells may represent the protective response of patients to UC-induced crypt inflammation.

### IBD–UC increases TCR, HLA and IL-17 gene signatures

We performed RNA-seq on colonic tissues from seven patients with UC with increased TCRγδ T cells in blood and crypt TCRγδ T cells (Fig. 1c and Supplementary Fig. 1b). We found these individuals show significantly increased *CD4*, *IL17*, *STAT5B*, *CISH, KIT*, *CTNNB1* and *STAT3* expression, coincident with reduced *IL23R*, *IL6, IL18, IL15* and *TCF7L2* versus controls (Fig. 1g). Gene set enrichment analysis (GSEA) further revealed that these patients with UC exhibit pronounced activation of IBD, JAK–STAT, IL-2, IL-15, IL-17, T_H_1, T_H_2, T_H_17, TCR, HLA-DR antigen processing and presentation, and CD34 progenitor pathways, coincident with suppressed pluripotency of stem cells (Fig. 1h–j and Supplementary Fig. 1g). These data suggest that an exaggerated JAK–STAT, TCR, IL-17 and HLA signaling pathway in UC affects ISC activity and progenitor regeneration, implicating increased interaction between TCRγδ T cells and ISCs in IBD–UC.

### Depleting STAT5 tetramer resulted in IEC hyperplasia and increased crypt T cells

**Fig. 2:**
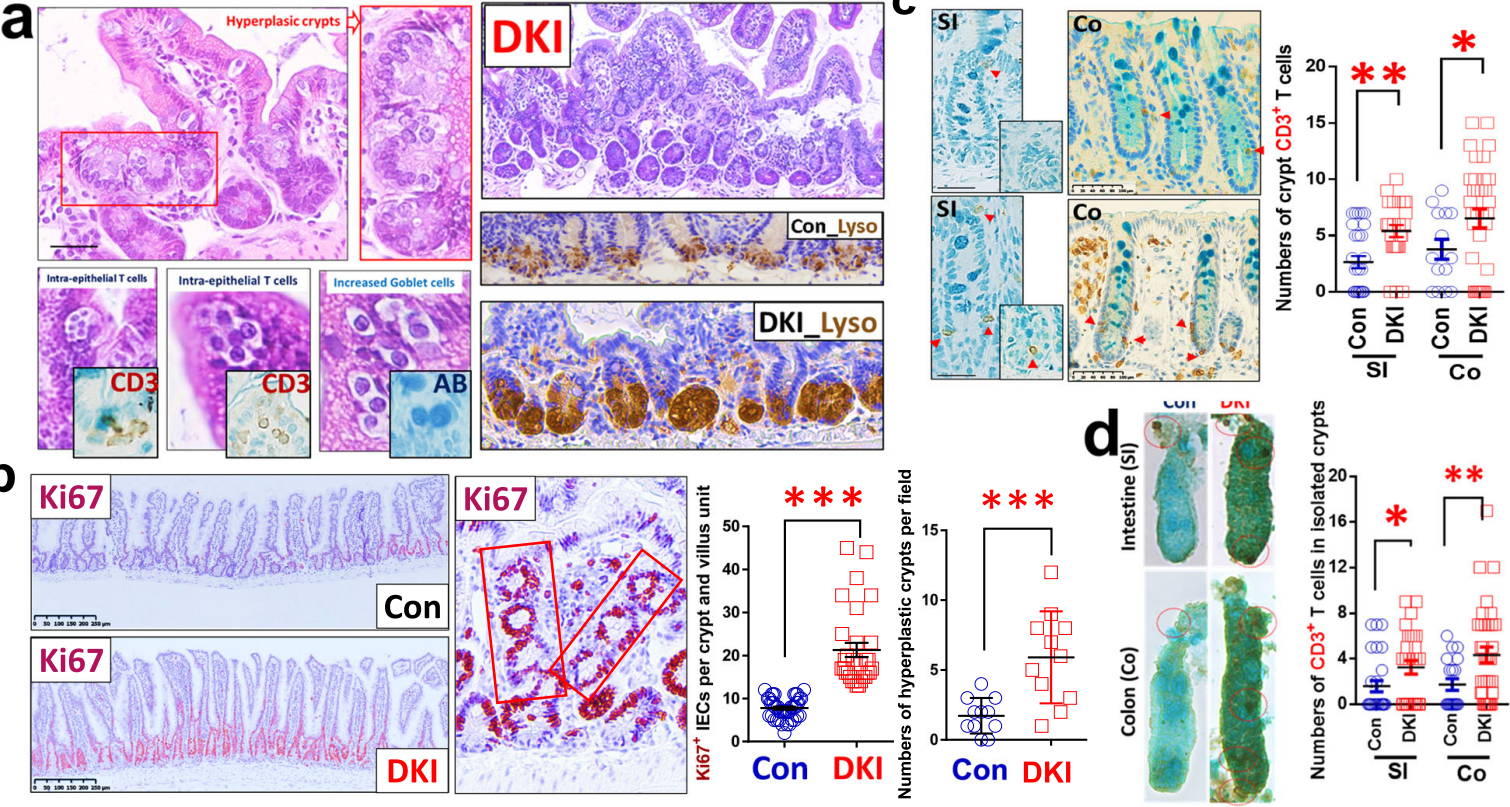
Depleting STAT5 tetramer led to IEC hyperplasia and increased CD3^+^ T cells in crypts. **a**, *Stat5a*–*Stat5b* DKI mice display hyperplastic intestinal crypts and increased intra-IEC lymphocytes, as shown with insert CD3 IH, goblet cells, as shown with insert AB staining, and lysozyme (Lyso)+ Paneth cells in the SI compared with WT controls (Con); *n* = 5 mice per group. **b**, DKI mice were killed, and SI sections were immunostained with anti-Ki67 antibodies. The number of Ki67^+^ IECs and hyperplastic crypts was counted. Results are expressed as mean ± s.e.m, ***P < 0.001 versus Con. **c**, SI or colonic (Co) sections were immunostained with anti-CD3 antibodies, and the numbers of CD3-positive T cells in the SI and Co crypts were counted (*n* = 5 mice per group). **d**, Isolated SI and Co crypts were immunostained with anti-CD3 antibodies and counterstained with Alcian blue (AB). The enumeration of intra-crypt CD3^+^ T cells is shown. Numbers of crypt T cells were counted in 100 isolated crypts per group. Results are expressed as mean ± s.e.m, **P < 0.01, ***P < 0.001 versus Con.

*Stat5a*–*Stat5b* DKI N-domain mutant mice form STAT5 dimers but not tetramers^28^ (Supplementary Fig. 2a). DKI mice have fewer intestinal CD8^+^ T cells and mucosal T_reg_ cells (CD4^+^CD25^+^ or Foxp3^+^) than WT littermate controls, leading to exaggerated colitis after adoptive T cell transfer^27,28^. These findings indicated that STAT5 tetramers are required for gut immune suppression. Our data further showed that STAT5 tetramer depletion induced hyperplastic proliferative IECs, hypertrophic crypts and increased goblet cell, PC, ISC and progenitor cell numbers (Fig. 2a,b and Supplementary Fig. 3a,b), displaying much bigger crypts and villi than WT control crypts and villi (Supplementary Fig. 3c). Strikingly, DKI mice showed a dramatic infiltration of intra-IEC lymphocytes and CD3^+^ T cells at crypt bases (Fig. 2c), which can be tightly adherent to crypt ISCs or IECs (Fig. 2d). These data suggest the direct role of crypt CD3^+^ T cells in the IEC hyperplasia and crypt hypertrophy. Next, we determined their effects on Lgr5 ISCs.

### Depleting tetrameric STAT5 in mice significantly increased Lgr5 regeneration

**Fig. 3:**
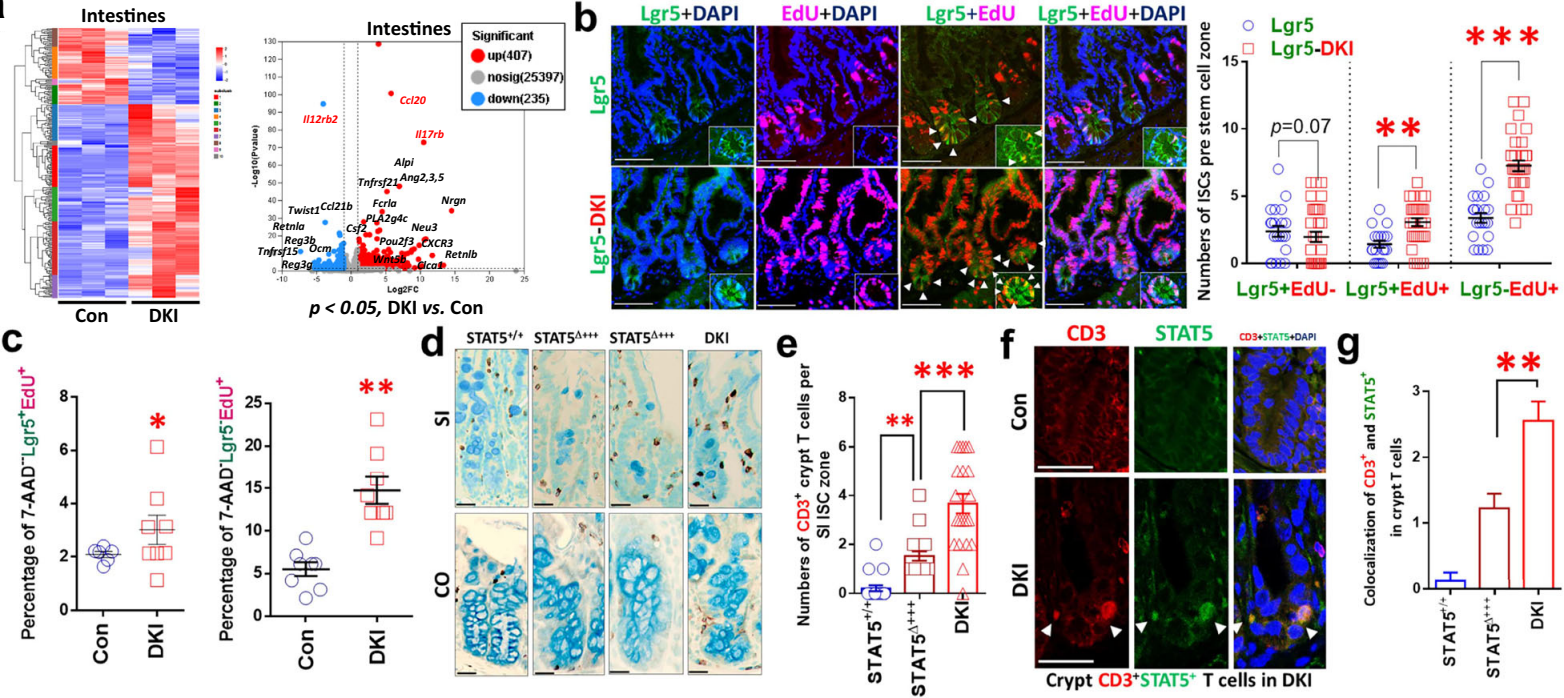
Depleting tetrameric STAT5 in mice significantly increased Lgr5^+^ Cell proliferation and CD3^+^ T cells in the crypts. **a**, SIs from *n* = 5 DKI and *n* = 5 WT control (Con) mice were dissected, and RNA was extracted for RNA-seq analysis. The heat map and volcano plot show 407 upregulated genes and 235 downregulated genes in DKI versus Con mice, with fold changes >2 and *P* values <0.05. DKI mice were crossed with *Lgr5Cre*ER (Lgr5) mice to mark LGR5^+^ ISCs with STAT5 tetramer depletion, and the mice were killed after an EdU intraperitoneal (i.p.) injection. **b**, SI sections were immunostained for Lgr5^+^EdU^+^ cells; the arrows show Lgr5 (green) and EdU (pink) cells. The number of Lgr5^+^EdU^−^, Lgr5^+^EdU^+^ and Lgr5^−^EdU^+^ cells was counted per ISC zone in each crypt. *P < 0.05, **P < 0.01, ***P < 0.001 versus Con. All results are expressed as mean ± s.e.m. **c**, SI crypts from DKI and Con mice were dissociated into single IEC cells and were gated and analyzed by Flow Jo to measure frequencies of 7-AAD^‒^Lgr5^+^EdU^+^ and Lgr5^‒^EdU^+^ crypt cells. *P < 0.05, **P < 0.01, ***P < 0.001 versus Con. **d**, **e**, SI from DKI, STAT5^Δ+++^ and Con (STAT5^+/+^) mice were dissected, and tissue sections were immunostained with anti-CD3 and counterstained with AB (**d**); crypt T cells were quantified as the number of crypt CD3^+^ T cells per crypt (**e**). **f**, **g**, SI tissue sections were immunostained with anti-CD3 and anti-STAT5 (**f**), and colocalization of CD3 (red) and STAT5 (green) was counted (**g**). The result is expressed as mean ± s.e.m.; *n* ≥ 50 crypts, **P < 0.01, ***P < 0.001 versus STAT5^Δ+++^.

Bulk RNA-seq and GSEA of SI tissue revealed no significant upregulation of genes associated with JAK–STAT, T_H_1, T_H_2 and T_H_17 cells; IL-17 signaling; or TCR pathways in DKI versus control mice (Fig. 3a). However, DKI mice show significant upregulation of *IL17rb*, *Tnfrsf21*, *Ccl20*, *Pla2g4c*, *Wnt5b* and *Pou2f3*, coincident with downregulation of the pro-inflammatory response genes *IL12rb2*, *Ccl21*, *Reg3b*, *Reg*3g, *Csf2* and *Twist1* (Fig. 3a), suggesting an exaggerated immune and repair response in the DKI mucosa. We then crossed DKI and *Lgr5Cre*ER mice to mark LGR5^+^ ISCs lacking STAT5 tetramers (Supplementary Fig. 2a). FACS using dissociated crypt cells (Supplementary Fig. 2b) and confocal IF show that these LGR5-DKI mice display significantly greater Lgr5^+^ and Lgr5^‒^ ISC proliferation (EdU^+^) (Fig. 3b,c) and LGR5^+^ ISC apoptosis (Supplementary Fig. 4a) than WT, indicating that depleting tetrameric STAT5 can significantly induce Lgr5 survival and regeneration. STAT5-overexpressing mice (*Rs26Cre*ER;cS5, STAT5^Δ+++^) have both STAT5 tetrameric and dimeric activation^25^, and the dimeric STAT5 activation can increase Lgr5^+^ ISC proliferation or regeneration^19,40^. Our data further reveal that STAT5^Δ+++^ mice exhibited significantly less numbers of crypt CD3^+^ T cells than DKI mice but still more than the number of crypt CD3^+^ T cells in WT, indicating that STAT5 tetramer depletion leads to greater crypt CD3^+^ T cells than STAT5 dimeric activation only and suggesting that STAT5 tetramer depletion may lead to the chromatin accessibility to control T cell influx into crypts or and increased interaction of T cells with crypt cells (Fig. 3d,e). Consistent with this notion, we also found that DKI can induce STAT5 overexpression, in addition to pYSTAT5 activation, in the crypt CD3^+^ T cells compared to control mice (Fig. 3f,g). These data confirm that the STAT5 tetramer inhibits T cell infiltration into the crypt compartment, possibly by epigenetic mechanisms. By contrast, STAT5 tetramer depletion can increase the number of CD3^+^ T cells entering the crypt bases by enhancing chromatin accessibility to genes controlling T cell differentiation and recruitment into crypts, other than STAT5 dimeric activation. Next, we determined the identity of crypt CD3^+^ T cells and their functions.

### Depleting tetrameric STAT5 in mice activated pYSTAT5 and upregulated IL-17A in crypt CD3^+^TCRγδ^+^ T cells

**Fig. 4:**
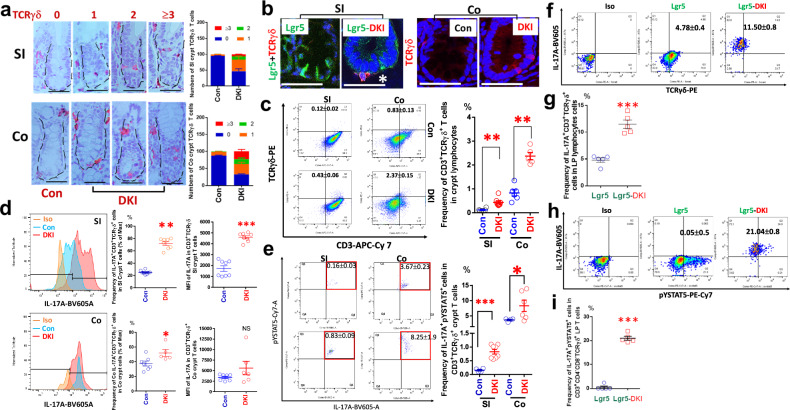
Depletion of tetrameric STAT5 in mice significantly increased crypt IL-17A^+^TCRγδ^+^ T cells. **a**, SI and colon (Co) sections from 5 Lgr5-DKI and 5 Lgr5 control (Con) mice were stained with TCRγδ IH. TCRγδ^+^ crypt T cells were quantified as ‘0’, no TCRγδ^+^ T cells, ‘1’, one TCRγδ^+^ T cell, ‘2’, two TCRγδ^+^ T cells, and ‘3’, three TCRγδ^+^ T cells. The results are expressed mean ± s.e.m. **b**, SI or Co sections were IF-stained to show Lgr5^+^ ISCs (green) and crypt TCRγδ^+^ T cells (red). The asterisk shows TCRγδ^+^ T cells within crypts. Images were captured with a confocal microscope. SI and Co crypts were isolated from 5 Lgr5-DKI and 5 Lgr5 Con mice and dissociated into single cells. The dissociated IECs were stained with anti-GFP, anti-CD3, anti-CD4 and anti-CD8, anti-TCRαβ and anti-TCRγδ, anti-pYSTAT5, and IL-17A FACS antibodies. **c**, Scatter graphs and frequency of SI or Co CD3^+^CD4^−^CD8^-^TCRγδ^+^ cells in the crypt lymphocytes are shown. The results are expressed as mean ± s.e.m. **P < 0.01 versus Lgr5-Con. **d**, IL-17A expression was determined as the frequency of SI or Co crypt IL-17A^+^CD3^+^TCRγδ^+^ T cells or the mean fluorescence intensity (MFI) of IL-17A in the SI or Co crypt CD3^+^TCRγδ^+^ T cells. Representative FACS scatter plots of isotype controls (iso), Lgr5-Con and DKI are shown. The results are expressed as mean ± s.e.m. **P < 0.01 versus Lgr5 Con. **e**, pYSTAT5 activation in the crypt IL-17A^+^TCRγδ^+^ T cells was determined as the frequency of pYSTAT5^+^IL-17A^+^ in the SI or Co crypt CD3^+^TCRγδ^+^ T cells. The representative scatter graphs of pYSTAT5 and IL-17A are shown, and the results are expressed as mean ± s.e.m. *P < 0.05 or ***P < 0.001 versus Lgr5-Con. **f**–**i**, The LP cells were gently dissociated using a MACS dissociator from four to five Lgr5-Con or Lgr5-DKI mice. The LP cells labeled for CD3, CD4, CD8, TCRγδ, pYSTAT5 and IL-17A were analyzed by FACS to identify CD3^+^CD4^−^CD8^-^TCRγδ^+^IL-17A^+^, CD3^+^CD4^−^CD8^−^TCRγδ^+^pYSTAT5^+^ (**f**) and CD3^+^CD4^-^CD8^-^TCRγδ^+^IL-17A^+^pYSTAT5^+^ (**h**) cell populations. Representative scatter plots showing the frequencies of CD3^+^CD4^-^CD8^**-**^IL-17A^+^TCRγδ^+^ T cells in LP lymphocytes (**g**) or IL-17A^+^pYSTAT5^+^ cells within TCRγδ^+^ LP T cells (**i**) are presented. Results are expressed as mean ± s.e.m. ***P < 0.001 compared with Lgr5-Con.

By combining IH and confocal IF, we found that crypt TCRγδ^+^ T cells are upregulated in the SI and colon of LGR5-DKI mice (Fig. 4a, b). These crypt TCRγδ^+^ T cells are located at the crypt base or in between Lgr5 ISCs (Fig. 4b). Consistently, FACS analysis using dissociated crypt cells showed that crypt CD3^+^TCRγδ^+^ T cells in the LGR5-DKI SI or colon are significantly greater than WT controls (Fig. 4c and Supplementary Fig. 4b). These data indicate that depleting tetrameric STAT5 can increase crypt CD3^+^TCRγδ^+^ T cells. Compared with WT, the frequency of pYSTAT5^+^ and/or IL-17A^+^CD3^+^TCRγδ crypt cells is increased in SI or colon of LGR5-DKI mice (Fig. 4d,e). Noticeably, the mean fluorescence intensity of intracellular IL-17A is increased in the SI crypt CD3^+^TCRγδ^+^ T cells in LGR5-DKI mice (Fig. 4d). In concomitant with upregulated pYSTAT5 and IL-17A in CD3^+^TCRγδ crypt cells upon depleting the tetrameric STAT5 (Fig. 4d,e), the number of Lgr5^+^ and Paneth cell niche cells is increased in the crypts (Figs. 2a and 3b). We then ask whether these crypt T cells are migrated from the LP. Using a gentle MACS dissociator to dissociate intestinal LP cells, we performed FACS analysis with CD3, CD4, CD8, TCRγδ, pYSTAT5 and IL-17A to identify CD3^+^CD4^−^CD8^−^TCRγδ^+^IL-17A^+^, CD3^+^CD4^−^CD8^−^TCRγδ^+^pYSTAT5^+^ and CD3^+^CD4^−^CD8^−^TCRγδ^+^IL-17A^+^pYSTAT5^+^ cells in LP. As shown in Fig. 4f–i, the frequency of LP IL-17A^+^TCRγδ^+^ cells and IL-17A^+^pYSTAT5^+^ TCRγδ^+^ T cells is significantly increased compared with control mice, suggesting that crypt TCRγδ T cells migrated from LP. In summary, these data indicate that depleting tetrameric STAT5 activates STAT5, leading to increased migration of CD3^+^TCRγδ^+^ crypt cells from the LP, which may promote Lgr5 ISC regeneration by secreting IL-17A^14^. These data also suggest a direct role for dimeric STAT5 activation, at the expense of suppressive tetrameric STAT5, IL-17A expression in crypt CD3^+^TCRγδ^+^T cells.

### Depletion of tetrameric STAT5 in mice protected ISCs from IR or colitis injuries and promoted de novo crypt regeneration

**Fig. 5:**
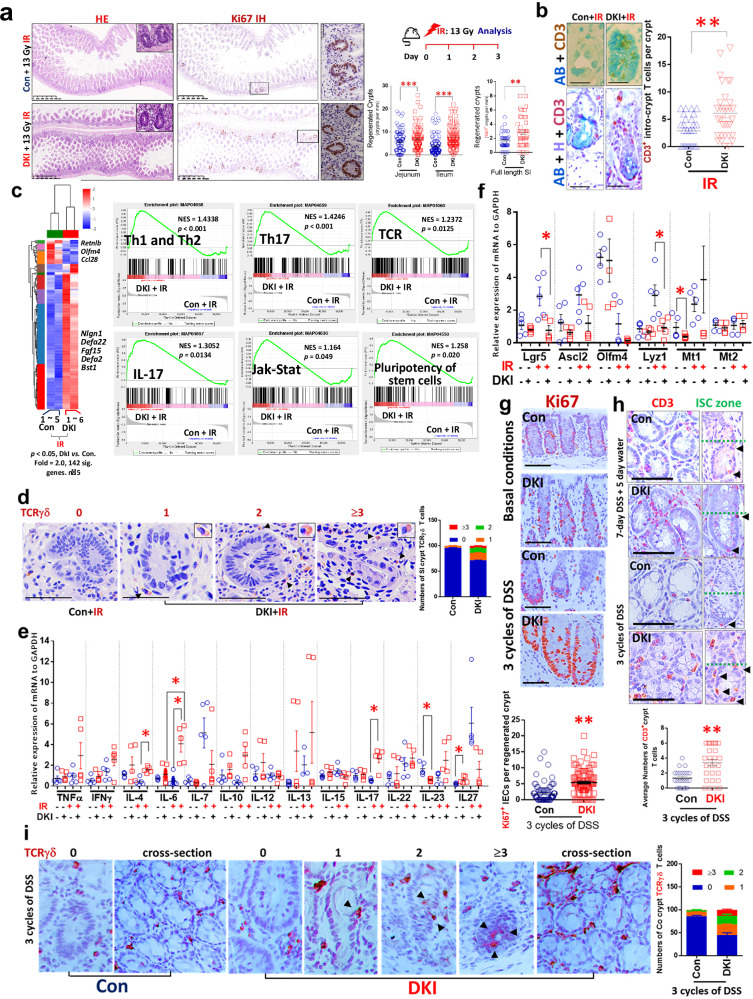
Depletion of tetrameric STAT5 in mice protected ISCs from IR or colitis injury and promoted de novo crypt regeneration and the crypt CD3^+^ or TCRγδ^+^ T cell niche. A total of six DKI and five control (Con) mice were treated with 13 Gy IR. **a**, At 3.5 days post IR, the mice were killed. SI sections were stained with HE and immunostained with anti-Ki67 antibodies; Ki67^+^ crypts were counted as regenerated. **b**, SI sections were immunostained with anti-CD3 antibodies and counterstained with AB; CD3^+^ crypt T cells were quantified. Results are expressed as the mean ± s.e.m.; *n* = 5 mice per group, **P < 0.01, ***P < 0.001 versus Con. **c**, RNA was extracted from SIs of a total of six DKI and five Con mice in two independent experiments and subjected to RNA-seq analysis. The heat map shows hierarchical clustering of 142 genes with ANOVA *P* < 0.05 and expression fold change >2 in DKI versus Con mice (*n* = 5 per group). GSEA shows activation of T_H_1, T_H_2 and T_H_17, as well as TCR, IL-17, JAK–STAT and pluripotency pathways. **d**, SI sections from five IR-treated DKI and five Con mice were immunostained with anti-TCRγδ IH. TCRγδ^+^ crypt T cells in the regenerative crypts were quantified as ‘0’, no TCRγδ^+^ T cells, ‘1’, one TCRγδ^+^ T cell, ‘2’, two TCRγδ^+^ T cells, and ‘≥3’, more than three TCRγδ^+^ T cells. The results are expressed as mean ± s.e.m. **e**, **f**, Mucosa tissues were isolated from DKI and Con mice with or without IR treatment. The total RNA was extracted, and mRNA levels of T_H_1, T_H_2 and T_H_17 cytokines (**e**), Lgr5 ISC, Paneth cell markers and Mt1 (**f**) were measured by qPCR. Results are expressed as the mean ± SEM; *n* = 5 mice per group, **P < 0.01, ***P < 0.001 versus Con. The same number of DKI and WT Con mice were treated with either three cycles of 2.5% DSS with a 10-day water recovery in between (i) or 7-day DSS followed by 5-day water recovery (ii), with *n* = 5 per group. **g**, **h**, The mice were killed. Colonic tissues were isolated, sectioned and stained with HE, Ki67 IH (**g**), and CD3 IH (**h**). The number of CD3- or Ki67-positive cells within the ISC stem cell zone was counted. The results were expressed as mean ± s.e.m.; *n* = 50 crypts, **P < 0.01 versus WT Con. Scale bar, 200 μm. **i**, Consecutive sections from five DKI and five WT Con mice with three cycles of DSS treatment were stained with TCRγδ IH. TCRγδ^+^ crypt T cells were scored as ‘0’ for no TCRγδ^+^ T cells, ‘1’ for one TCRγδ^+^ T cell, ‘2’ for two TCRγδ^+^ T cells, and ‘3’ for three TCRγδ^+^ T cells. The results are expressed as mean ± s.e.m. Representative cross-sections showing TCRγδ^+^ T cell staining are displayed.

To further elucidate the role of STAT5 tetramerization in forming the crypt TCRγδ T cell niche and ISC regeneration. We treated DKI and WT control mice with 13-Gy ionizing irradiation (IR). The HE staining and IH for Ki67 in DKI and WT mice treated with IR revealed significantly increased regenerated crypts in DKI mice compared to controls, which may be indicative of increased ISC regeneration in DKI animals (Fig. 5a). Notably, we also detected a robust increase in crypt CD3^+^ T cells and goblet cells in DKI crypts (Fig. 5b and Supplementary Fig. 5a), suggesting a positive role for crypt T cells in the regulation of ISC regeneration in vivo. RNA-seq and GSEA analyses further show that IR-treated DKI mice exhibit activated JAK–STAT and increased Th1, Th2, and Th17 cell differentiation, stem cell pluripotency pathways, cytokine–cytokine receptor interactions, and antigen processing and presentation (Fig. 5c). Strikingly, numbers of the crypt containing TCRγδ^+^ T cells in DKI mice were significantly higher than TCRγδ^+^ T cells in WT (Fig. 5d). qPCR shows that the upregulated *IL-4*, *IL-6*, *IL-17* and *IL-27*, in contrast to reduced *IL-23*, *Lgr5* and Metallothioneins (*Mt1*) by comparing DKI with Con (Fig. 5e,f). These data suggest enhanced crosstalk between TCRγδ^+^ T cells and antigen-presenting cells or stem cells in DKI mice. Consistently, using two DSS-induced colitis models (Supplementary Fig. 5b(i),(ii)), particularly by three cycles of 5-day DSS following 16-day water recovery specific for apoptosis-induced colonic ISC regeneration^19,43^ (Supplementary Fig. 5c), we found that depleting STAT5 tetramers significantly increased the regeneration of nascent crypts adjacent to ulcers compared with WT controls (Fig. 5g), which is coincident with elevated colonic crypt CD3^+^ T cells (Fig. 5h). In those regenerated colon crypts, IH shows that colonic crypt TCRγδ^+^ T cells are increased in DKI mice (Fig. 5i), which is consistent with IBD–UC colon crypts, where more crypt lymphocytes and TCRγδ^+^ T cells appeared (Fig. 1c,d and Supplementary Fig. 1e). These data suggest that the crypt TCRγδ^+^ T cells act as a niche to assist colonic ISC regeneration.

### Tetrameric STAT5-deficient organoids exhibited reduced growth under IR exposure compared with the WT control group

**Fig. 6:**
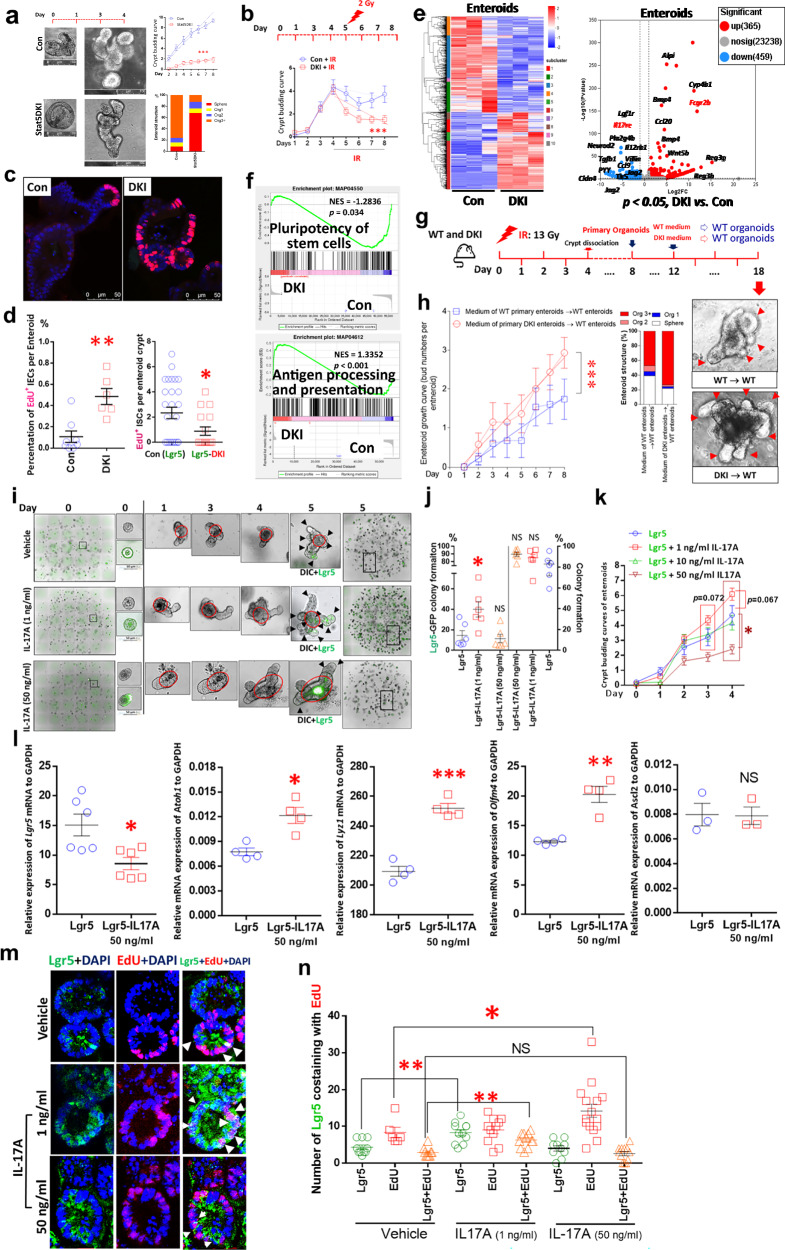
Tetrameric STAT5-deficient organoids exhibited reduced growth under IR exposure compared with the control group. Enteroids were differentiated from the SI crypts of DKI and control (Con) mice. **a**, A subset of these enteroids was used to quantify proliferation and multiplicity. Results are expressed as the mean ± s.e.m.; >10 enteroids per group were traced and counted, ***P < 0.001 versus Con. **b**, The remaining enteroids were exposed to 2-Gy IR for 10 min on day 5 and allowed to differentiate for another 4 days. Organoid budding curves were generated from *n* = 10 enteroids per group, ***P < 0.001 versus Con + IR. **c**, Enteroids from Lgr5-DKI and *Lgr5Cre*ER mice were differentiated for 14 days with EdU added on day 10. **d**, EdU^+^ IECs or Lgr5^+^ ISCs were analyzed; >10 enteroids per group were counted, *P < 0.05, **P < 0.01 versus Con. **e**, After passage, enteroids were differentiated for 7 days, and RNA was extracted for RNA-seq analysis. The heat map and volcano plot show differentially expressed genes with fold change >2.0 and *P* < 0.05; *n* = 3 mice per group. **f**, GSEA reveals upregulation of TCR activation, stem cell pluripotency and antigen-processing and antigen-presentation pathways. **g**, DKI and Con mice were treated with 13 Gy IR. At 3.5 days post IR, the mice were killed. Primary enteroids were differentiated from the SI crypts of DKI and Con mice for 8 days. The medium from the primary intact crypt-derived enteroids of DKI and WT Con was transferred to the WT Con enteroids to coculture for 6 days, and the number of crypt budding and enteroids multiplicity were determined. **h**, The variance analysis was used to analyze the difference between the two groups treated with the transferred medium from DKI and WT Con. Results are expressed as the mean ± s.e.m., *P < 0.05, **P < 0.01 versus Con. All data presented in the organoid culture are representative of at least three repeated experiments. **i**–**k**, The enteroids were derived from intact Lgr5 crypts dissociated from Lgr5CreER mice. The enteroids were stimulated with IL-17A (1, 10 or 50 ng/ml) for 6 days. Representative GFP or GFP + DIC images of enteroids or IL-17-treated samples are shown from day 0 to day 5 (**i**). Lgr5 colony formation or colony growth in the 1 ng/ml or 50 ng/ml-treated enteroids (**j**), and budding curves of enteroids treated with 1, 10 or 50 ng/ml (**k**) were measured from 100 to 200 Lgr5^+^ or Lgr5^−^ intact crypt-derived enteroids. Results are expressed as the mean ± s.e.m, *P < 0.05, versus Lgr5-Con. **l**, The enteroids on day 5 were collected, and total RNA was extracted. The expression levels of Lgr5, Atoh1, Lyz1, Olfm4 and Ascl2 were quantified using qPCR. Results are shown as mean ± s.e.m., with no significant differences (NS), *P < 0.05, **P < 0.01 or ***P < 0.0001 compared with Lgr5-Con, *n* = 4–6. **m**, **n**, The enteroids treated with 1 ng/ml or 50 ng/ml on day 5 were incubated with EdU, then collected and fixed with paraformaldehyde (PFA). Sections of 10-μm thickness were stained with anti-GFP and EdU IF (**m**). Lgr5^+^, EdU^+^ or Lgr5^+^EdU^+^ organoid cells were quantified as the number per organoid crypt; scatter plots are shown (**n**). Results are expressed as the mean ± s.e.m., with no significant difference (NS), *P < 0.05 or **P < 0.01 compared with Lgr5-Con, *n* = 4–6.

In contrast to in vivo data, DKI enteroids generated from DKI mice exhibit reduced ISC proliferation and increased IEC hyperplasia compared with WT enteroids, manifested by reduced budding rate and increased number of spheroids in DKI enteroids (Fig. 6a). Proliferation of DKI enteroids is inhibited by 2-Gy IR to a greater extent than WT enteroids (Fig. 6b), indicating that depleting STAT5 tetramer decreases ISC proliferation and regeneration in vitro in an intrinsic manner. Intriguingly, confocal IF analysis revealed that enteroids differentiated from LGR5-DKI mice have fewer EdU^+^ ISCs at crypt bases and more EdU^+^ IECs in the villi and TA zone (Fig. 6c,d and Supplementary Fig. 6a), suggesting that STAT5 tetramer depletion increases progenitor hyperplasia. Consistent with these data, RNA-seq showed reduced stem cell pluripotency and enhanced antigen-processing and antigen-presentation pathways in DKI enteroids (Fig. 6e,f). Notably, the culture medium from the primary enteroids directly differentiated from IR-treated DKI crypts adhered by CD3^+^ T cells, exhibited the enhanced stimulation of WT enteroids growth, manifested by increased budding rate and reduced the number of spheres (Fig. 6g,h and Supplementary Fig. 6b). These data suggest that the in vivo effects of STAT5 tetramer depletion on Lgr5 ISC regeneration are due to niche cells like crypt TCRγδ^+^ cells. Together, these data suggest that STAT5 tetramer depletion drives ISC regeneration by promoting crypt T cell niche formation, thereby protecting Lgr5^+^ ISCs from injury. STAT5 tetramerization plays opposing roles in IECs and T cells, and DKI mediates ISC regeneration in vivo, possibly via STAT5 activation in the crypt T cells.

### IL-17A promoted crypt IEC regeneration in a biphasic, dose-dependent manner

Our results revealed that depleting tetrameric STAT5 increases IL-17A expression in TCRγδ T cells at both crypt and LP (Fig. 4d,f). IL-17A has a dual, context-dependent role in ISCs, serving as a key promoter of tissue repair by inducing Lgr5^+^ ISCs to differentiate into secretory cells (goblet, Paneth, tuft) through the transcription factor ATOH1^14^. Inhibiting IL-17A secretion worsens IBD severity^44^. However, during chronic inflammation, excessive IL-17 signaling can paradoxically promote abnormal proliferation or apoptosis of ISCs, highlighting its complex interactions with intestinal homeostasis and disease^45^. Still, it remains unclear at what doses or timing IL-17A can directly act on Lgr5^+^ ISCs or on other cell types during differentiation. To address these questions, we tested different doses (1, 10 and 50 ng/ml) of IL-17A on intact crypt-derived organoids, examining Lgr5 colony-forming efficiency, budding index, ATOH1/LGR5 programs and EdU incorporation. Using enteroids derived from intact Lgr5 crypts dissociated from *Lgr5Cre*ER mice, we stimulated the enteroids with IL-17A (1, 10 or 50 ng/ml). As shown in Fig. 6, 1 ng/ml of IL-17A increases crypt budding of Lgr5 enteroids and Lgr5 ISC proliferation compared to vehicle control, indicated by colony formation of Lgr5-GFP buds and the number of Lgr5^+^EdU^+^ ISCs (Fig. 6j,k,m,n). By contrast, 50 ng/ml of IL-17A promotes spheroid formation and organoid IEC proliferation, as demonstrated by organoid budding in Fig. 6i,k, multiplicity in Supplementary Fig. 6c and EdU staining in Fig. 6m,n, after 5-day induction with IL-17A. Consistently, qPCR and IF results show that 50 ng/ml of IL-17A can significantly increase Atoh1, Lyz1 and Olfm4 expression as well as the number of Lyz1^+^ organoid IECs and Lgr5^−^EdU^+^ spheroid IECs while decreasing Lgr5 and Ascl2 compared with vehicle control (Fig. 6l,n and Supplementary Fig. 6d,e), indicating that higher doses of IL-17A can halt Lgr5 ISC self-renewal and promote differentiation toward secretory lineages. However, using intact crypt-derived enteroids treated with IL-17A, our data also suggest that IL-17A might act on Lgr5^+^ or Olfm4^+^ ISCs differently or increase the switch of Lgr5 into Olfm4 ISCs that can repair the apoptotic IECs^46^, as evidenced by 50 ng/ml of IL-17A inhibiting Lgr5 while increasing Olfm4 expression in enteroids. By integrating scRNA-seq, GSEA and trajectory analysis, we next determined how STAT5 tetramer depletion enhances the migration of crypt TCRγδ^+^ T cells into the ISC compartment and their differentiation into niche cells, thereby facilitating ISC regeneration.

### STAT5 tetramer depletion increased crypt T cell interaction with ISCs at a single-cell level, propelling them to form a healing cell population

**Fig. 7:**
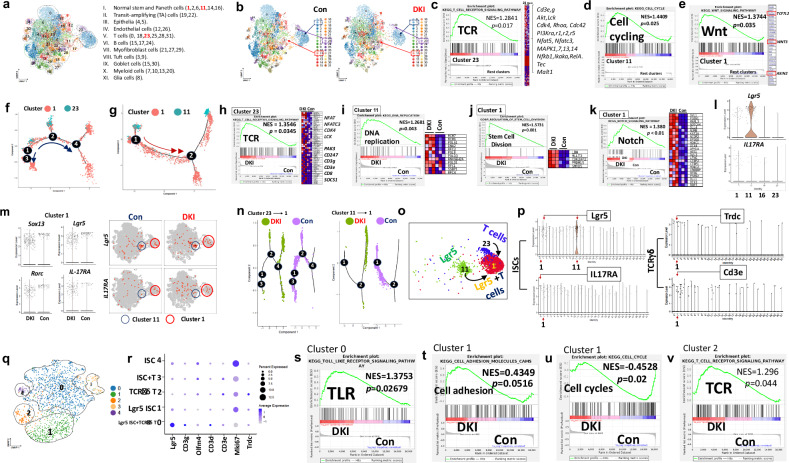
STAT5 tetramer depletion increased the interaction of crypt T cells and ISCs at a single-cell level. SI mucosal cells from three DKI and three WT control (Con) mice were dissociated into single cells, and 13,000 single cells per sample were analyzed by scRNA-seq. **a**, **b**, A total of 32 populations of intestinal cells were annotated and clustered into 11 types for both DKI and Con mice. **a** Intestinal cells are annotated into 32 populations of cells. **b** Thirty-two populations respectively in Con or DKI mice. Arrows show population 1, 11, or 23 in Con and DKI mice. **c**, GSEA analyses reveal that the TCR signaling pathway in cluster 23 is markedly activated compared with the other clusters, along with significant upregulation of *CD3*, *PI3K*, *NFκB* and *MAPK* genes. **d**, **e**, Compared with the rest of the clusters, cell cycling signaling in cluster 1 is significantly upregulated (**d**), and Wnt signaling is elevated with robustly activated *TCF7L2*, *WNT3* and *AXIN2* genes (**e**). The normalized enrichment score (NES), and adjusted *P* values are shown. Trajectory analysis was done by cell states and shows the roots of cell differentiation and maturation. **f**, T cells in cluster 23 were driven to differentiate into T cells in cluster 1 via sequential cell states 1–2–4 or 1–3. The arrows show the direction of T cell differentiation. **g**, Stem cells in cluster 11 were driven to mature toward the stem cells in cluster 1 by cell states, 1–2. The arrows indicate the direction of stem cell maturation. **h**–**k**, Compared with WT Con, GSEA analyses reveal that DKI exhibited significantly activated TCR signaling in the T cell population (cluster 23) (**h**), increased DNA replication in the stem cell population (cluster 11) (**i**), enhanced stem cell division (**j**) and elevated Notch activation (**k**) in the stem cell and T cell mixed population (cluster 1); NES and adjusted *P* values are shown. **l**, Compared with the rest of the clusters, *Lgr5* and *IL-17RA* genes are highly enriched in clusters 11 and 1. **m**, DKI exhibits increased *Sox13*, *Rorc*, *Lgr5* and *IL-17RA* genes in cluster 1 compared with cluster 1 in WT Con. Representative violin graphs and scatter graphs are shown. **n**, Pseudotime trajectory analyses reveal that the cell states in clusters 23 or 11 in DKI progress toward cluster 1 more quickly and more strongly than in WT Con, suggesting that DKI increases the transition of cluster 11 or 23 toward cluster 1 more than WT Con. **o**, Depleting STAT5 tetramers promotes the formation of an Lgr5 ISC and a T cell population with vigorous healing capacity. **p**, Using Loupe analysis to analyze 32 populations, Lgr5 and IL17RA are highly enriched in cluster 1, suggesting that it contains a large number of ISCs. Trdc and Cd3e are highly enriched in cluster 1, suggesting that it contains a large number of ISCs and TCRγδ T cells. **q**, Cluster 1 was further separated into five populations: 0–4. **r**, The bubble plot shows the expression of Lgr5, Cd3e, Olfm4, Cd3d, Cd3e, MKi67 and Trdc in different populations separated from cluster 1. **s**–**v**, Compared with WT Con, GSEA analyses reveal that DKI significantly activated TLR in population 0 (**s**), significantly upregulated cell adhesion pathway in DKI compared to Con (**t**), significantly upregulated cell cycle pathway in DKI compared to Con (**u**) and the TCR pathway in population 2 (**v**). NES and adjusted *P* values are shown.

Although ISCs were reported to crosstalk with T cells through MHC–TCR or E-cadherin-integrin αEβ7 interactions, it is not known how ISCs and T cells interact in the context of the deregulated cytokines that are often aberrantly expressed during mucosal inflammation^11,15,17^. To address this question, we performed scRNA-seq on dissociated SI mucosal cells and identified 32 SI cell populations (Fig. 7a,b, and Supplementary Fig. 7a). Of these, population 23 is characterized by a TCR gene signature and defined as the T cell population. Population 11 is characterized by an Lgr5 ISC gene signature with enhanced cell cycling and is defined as the ISC population. Population 1 is characterized by Lgr5 ISC and TCR cell gene signatures with activated Wnt and T cell signaling, and is defined as the ISC + T cells population (Fig. 7c–e and Supplementary Fig. 7b,c). The cells in cluster 1 have a potent regenerative capacity with enhanced TGF-β signaling and innate immune response (Supplementary Fig. 7d,e). Trajectory analysis reveals that T cells in cluster 23 are driven to differentiate toward the T cells in cluster 1, suggesting that the T cells in cluster 1 have more robust or active functions than the T cells in cluster 23 (Fig. 7f). Stem cells in cluster 11 are driven to mature toward the stem cells in cluster 1, suggesting that the cells in cluster 1 are more active in cell division, proliferation, and regeneration than the stem cells in cluster 11 (Fig. 7g). Importantly, compared with WT control, GSEA analysis revealed that depleting STAT5 tetramer leads to increased TCR pathway and reduced JAK–STAT activation in the T cell population (cluster 23; Fig. 7h and Supplementary Fig. 7f), increased DNA replication in the Lgr5 ISC population (cluster 11; Fig. 7i) and elevated stem cell division, Notch and TLR signaling while reduced allograft rejection and cell adhesion signaling in the ISC and T cell mixed population (cluster 1; Fig. 7j,k and Supplementary Fig. 7g–i). These data indicate that depleting STAT5 tetramer can enhance the regenerative capacity in cluster 1. Intriguingly, *IL-17RA* is increased in cluster 1 besides *Lgr5* (Fig. 7l). Compared with cluster 1 in WT controls, TCRγδ cell regulatory genes^47^, such as *Sox13*, *Rorc* and *Cd3e*, are increased, and the genes regulating ISCs^14^, such as *Lgr5*, *Olfm4* and *IL-17RA*, are upregulated in cluster 1 in DKI, suggesting an enhanced ISC function in cluster 1 possibly via IL-17RA (Fig. 7m and Supplementary Fig. 7j). Trajectory analysis revealed that depleting STAT5 tetramer promotes the transition of T cell cluster 23 or Lgr5 ISCs in cluster 11 toward the T cell and Lgr5 ISC populations in cluster 1 (Fig. 7n). Taken together, we demonstrate that STAT5 tetramer depletion enhances IL-17A action of TCRγδ T cells on Lgr5^+^ ISCs, possibly by activating Notch signaling in T cells and Wnt signaling in ISCs. This effect could cooperate with Paneth cells to promote Lgr5 ISC regeneration and repair during mucosal inflammation with the deregulated cytokines (Fig. 7o).

To demonstrate our hypothesis, we further analyzed cluster 1 and examined gene expression in Lgr5 cells and T cells using specific markers: Lgr5 and IL-17RA for crypt Lgr5 cells and Cd3e and Trdc for TCRδ T cells. Our results confirm that cluster 1 is a mixed population including Lgr5 cells and TCRδ T cells (Fig. 7p). We divided cluster 1 into five subpopulations (clusters 0, 1, 2, 3 and 4; Fig. 7q). We identified that cluster 0 represents Lgr5 ISCs and TCRγδ T cells, marked by Lgr5, Cd3d and Trdc as shown in Fig. 7r. TLR signaling in DKI cluster 0 is significantly upregulated compared with control, suggesting increased healing activity in DKI (Fig. 7s). Cluster 1 is defined as Lgr5 ISCs and characterized by Lgr5. Cell adhesion in DKI cluster 1 increases, whereas cell cycling decreases, suggesting elevated Lgr5 and T cell adhesion tendency in cluster 1 (Fig. 7t,u). Cluster 2 is characterized by Trdc and increased TCR signaling, indicating activated T cell function (Fig. 7v). We next determine how crypt TCRγδ cells are differentiated and migrated from the mucosa upon STAT5 tetramer depletion.

### Tetrameric STAT5-deficient mice displayed reduced STAT5 binding to the *Mt1* gene, thereby relieving the repression of TCRγδ cell differentiation and migration into crypts

**Fig. 8:**
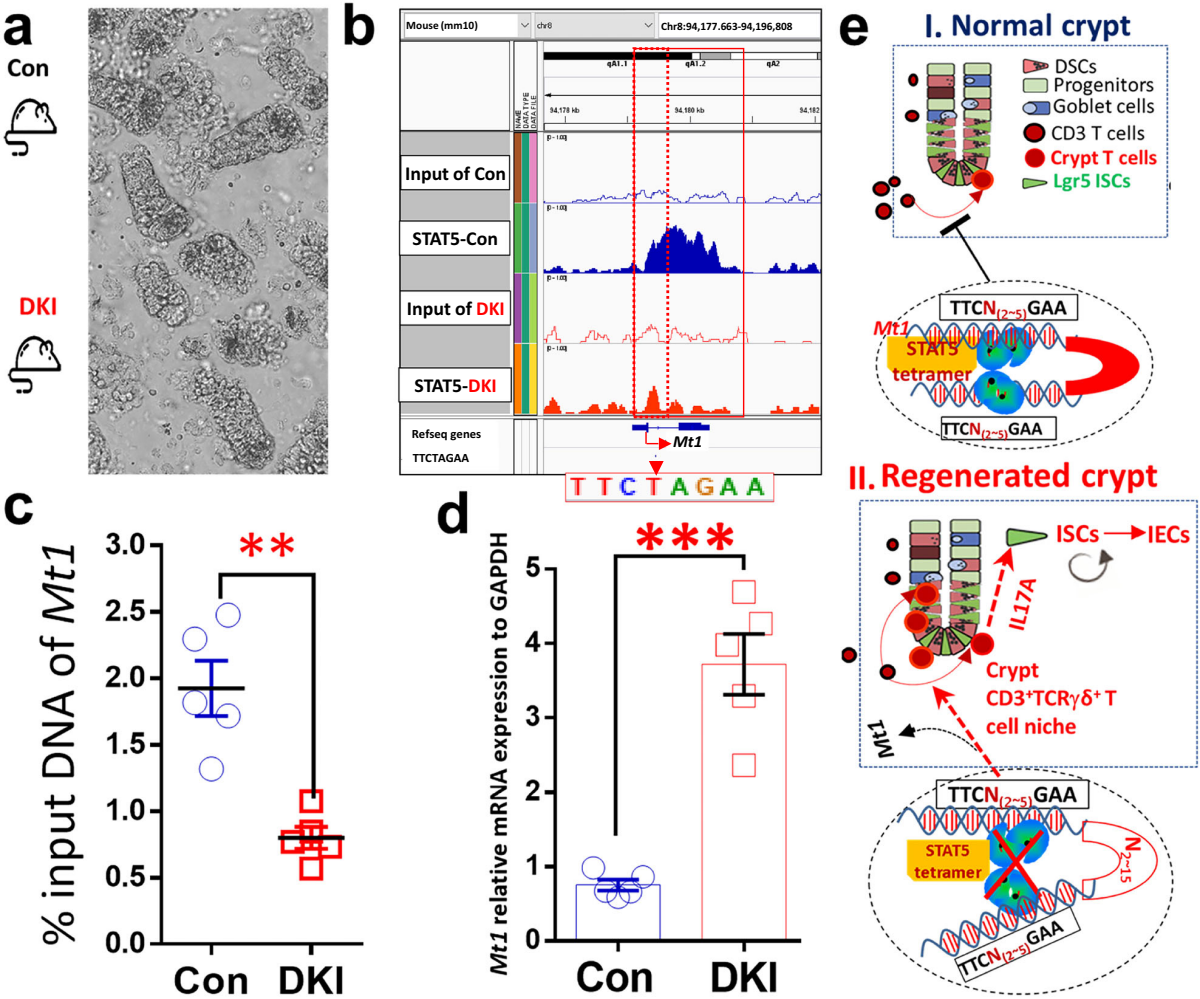
Depleting STAT5 tetramers decreased STAT5 binding to the *Metallothionein 1* (*Mt1*) gene and released the repression of TCRγδ cell differentiation. **a**, SI from three DKI and three control (Con) mice were dissected, and intestinal crypts were dissociated. **b**, The nuclear proteins and DNA complex were extracted from 2000 crypts, cross-linked and then immunoprecipitated with total STAT5 antibodies. Libraries for next-generation sequencing were also prepared and sequenced with a HiSeq 2500 instrument. **c**, qPCR was used to measure the GAS motif (TTCTAGAA) at the *Mt1* locus using nuclear protein–DNA complexes extracted from the crypts of DKI and Con mice. *N* = 5 mice per group, **P < 0.01 versus Con. **d**, The RNA from the dissociated crypts was extracted and analyzed by qPCR to determine *Mt1* expression. The relative expression of *Mt1* is quantified in the DKI and Con groups. Results are expressed as the mean ± s.e.m., **P < 0.01 versus Lgr5-Con, *n* = 5. **e**, Diagram description: STAT5 tetramers inhibit T cell influx into crypt bases (i) under normal conditions. During regeneration, depletion of STAT5 tetramers may lead to the formation of a crypt TCRγδ T cell niche, promoting ISC regeneration and IBD healing by increasing IL-17A interactions with IL-17RA on Lgr5 ISCs (ii).

Metallothionein 1 (MT1) proteins can suppress T regulatory type 1 (T_R_1) and T_H_17 cell differentiation while regulating CD4^+^ T cell differentiation toward T_reg_ cells^48–50^. Interestingly, MT1 can be released into the extracellular matrix to stimulate inflammation. Although M1 regulation and function are poorly understood, *Mt1* can be mainly expressed in T cells^49^. ChIP sequencing and qPCR analyses of SI crypts (Fig. 8a) showed that STAT5 highly binds the *Mt1* locus under standard conditions. By contrast, STAT5 tetramer depletion significantly decreases STAT5 binding on *Mt1* locus (Fig. 8b,c), indicating that the reduced occupancy of STAT5 on *Mt1* induced by STAT5 tetramer depletion releases the STAT5 repression of *Mt1* activity. Interestingly, H3K4Me3 recruited by STAT5 tetramer depletion was slightly upregulated on the *Mt1* promoter compared with input controls (Supplementary Fig. 8), suggesting that the repressed *Mt1* activity induced by STAT5 tetramers is attributed to a suppressive chromatin that could be formed by a long-range interaction of the *Mt1* promoter with enhancers or silencers. qPCR on crypt RNA extracted from control and DKI samples revealed that *Mt1* expression is significantly higher in DKI than in controls (Fig. 8d). These data suggest that, upon STAT5 tetramer depletion, STAT5 binding to *Mt1* genes in crypt T cells decreases, leading to increased *Mt1* levels that promote T cell migration into the crypt ISC compartment.

## Discussion

It is largely unknown how ISC self-renewal and differentiation are regulated in response to intestinal inflammation, leading to a lack of effective treatments for IBDs. In this Article, we investigate a new type of crypt T cells inside the ISC zone upon STAT5 dimerization activation. Interestingly, these crypt T cells can be retained within the ISC zone to act as niche cells, at the expense of STAT5 tetramer depletion, which generates a suppressive chromatin marked by H3K27me3. At the same time, STAT5 dimerization activation must remain simultaneously (Fig. 8e). Our data show that this new type of crypt T cells can protect ISC regeneration from IR or colitis injury. Mechanistically, depletion of tetrameric STAT5 relieves *Mt1*-mediated repression of TCRγδ cell differentiation and drives TCRγδ cells to migrate from LP into and retain in the ISC zone. The IL-17 secreted by crypt TCRγδ cells can enhance Lgr5 ISC regeneration and IEC repair by binding IL17-RA on ISCs (Fig. 8e). Importantly, TCRγδ^+^ can be found increased at the colonic crypts of IBD–UC, suggesting that this new type of crypt TCRγδ T cells could be targeted for improving ISC injury of IBD–UC.

Based on our data from spatial localization at homeostasis or during IR-induced intestinal, IBD or DSS-induced colonic injuries, we define this population of crypt TCRγδ T cells as follows: these crypt T cells are TCRγδ cells located at the crypt bases, including the TA cell zone, considering that Lgr5 cells can expand under inflammation, stress or insults (Figs. 1d and 4a and Supplementary Fig. 1e). They can be positioned between Lgr5 ISCs (Fig. 4b) or around the crypt bases (Figs. 4a and 5i) and are presumably in direct contact with ISCs. Biologically, after induction by inflammation or tetrameric STAT5 depletion, these CD3^+^CD4^−^CD8^-^TCRγδ^+^ crypt T cells exhibit increased STAT5 expression (Fig. 1d), nuclear localization (Fig. 3f) and produce or express high levels of IL-17A (Fig. 4d). These TCRγδ T cells may display active Notch signaling (Fig. 7k), T cell chemotaxis (Supplementary Fig. 7c), TGF-β signaling (Supplementary Fig. 7d) and pathways related to cell adhesion (Supplementary Fig. 7h). An increase in the influx of crypt TCRγδ T cells from LP could result from the release of STAT5-mediated repression of *Mt1* and increased *Mt1* expression (Fig. 8b–d). Interestingly, API5 is a protein secreted by intra-IEC TCRγδ T cells that acts as a vital survival factor for Paneth cells. It helps preserve the intestinal barrier by preventing the loss of Paneth cells, especially in individuals with a genetic susceptibility to Crohn’s disease (for example, those with an ATG16L1 risk allele)^51^. We costained crypt T cells for TCRγδ and API5 in control and DKI mice, as well as in healthy controls, IBD (UC) and IBD (Crohn’s disease). We found that a few crypt TCRγδ T cells express API5 in control mice, with partial colocalization with TCRγδ staining in IBD–UC (Supplementary Fig. 1h). We observed reduced API5 levels in IBD (Crohn’s disease) and increased API5 expression in crypt TCRγδ cells in IBD (UC) (Supplementary Fig. 1h), suggesting a potential role for API5 in the influx of TCRγδ T cells into the crypt base during UC. However, DKI mice show increased TCRγδ or API5, and TCRγδ-API5 colocalization is not prominent in crypt T cells (Supplementary Fig. 4c). Given that these API5 data are preliminary and require validation in a larger cohort of patients with IBD, we will investigate whether API5 colocalizes with crypt TCRγδ T in the future .

Human TCRγδ T cells are found in three locations in the intestinal and colonic mucosa: (1) LP (LP TCRγδ), (2) intra-epithelial (IEL TCRγδ) and (3) Peyer’s patches (PP TCRγδ)^52^. These TCRγδ T cells play critical immune-surveillance roles, suppressing exaggerated mucosal inflammation and eradicating malignant crypt ISCs. However, they can also mediate IECs to repair injury^53,54^. A recent publication further reported that intra-IEC TCRγδ T cells can limit IBD progression^55^. Intriguingly, epigenetic transcriptional repression mediated by STAT5 dimerization activation or STAT5 tetramerization was shown to be critical for the regulation of intra-IEC TCRγδ T cell functions or lineage differentiation by IL-2, IL-7 and IL-15 in response to gastrointestinal inflammation^37,56,57^. We found that depleting STAT5 tetramer can increase the number of crypt TCRγδ T cells (Fig. 4a–c) and promote IL-17 secretion by activating pYSTAT5 (Fig. 4d–i). Thus, depleting STAT5 tetramer leads to the generation of special crypt IL-17^+^TCRγδ T cells that have been demonstrated to improve mucosal healing by inducing Lgr5 ISCs to differentiate into secretory cells^14^. DKI SI mucosa under basal conditions expresses significantly increased *Wnt5b* and *Tnfrsf21*, in contrast with reduced mucosal *IL-23*, *IL-12*, *Reg3b*, and *Reg3g* compared to Con mice (Figs. 3a and 5e). Our IR experiment exhibited increased mucosal *IL-4*, *IL-6*, and *IL-17* in DKI mice compared to Con (Fig. 5c,e). Consistently, the bulk RNA-seq analysis shows the upregulated T_H_1, T_H_2 and T_H_17 cell differentiation, IL-17, Jak–Stat, TCR and stemness pathways whereas reduced *Lgr5*, *Ascl2* and *Lyz1* (Fig. 5c,e), which indicated the enhanced mucosal repair in the DKI mice, possibly involved by both active Lgr5 and facultative ISCs^58^ (Fig. 7m,r and Supplementary Fig. 7j). Notably, the nascent crypts have dramatically upregulated CD3^+^ or TCRγδ T cells (Fig. 5b,d), which could be the sources of healing cytokines such as IL-17, TGFβ, IL-10 and Wnt5b (Fig. 5c). We have shown IL-17A has a biphasic effect on ISC regeneration by targeting the different ISC populations (Fig. 6i–n). Using Lgr5–GFP-ki67 YFP enteroids, further research is ongoing to determine which types of ISCs IL-17 targets. Importantly, the culture medium from the primary organoids differentiated from IR-treated DKI crypts adhered with T cells, exhibited the enhanced stimulation of WT enteroids growth (Fig. 6g,h and Supplementary Fig. 6b), indicative of the effects of crypt TCRγδ T cells on ISC proliferation.

We further tested the major interactomes involved in the ISC regeneration driven by the increased crypt TCRγδ T cells. The scRNA-seq showed that amongst 32 intestinal cell populations, the cluster 23 showed increased TCR gene signatures (Fig. 7c). By contrast, the cluster 11 exhibited increased cell cycling signaling, suggesting the active Lgr5 cells (Fig. 7d). Strikingly, the cluster 1 has both TCR and Wnt gene signatures, suggesting a mixed population of ISC and T cells (Fig. 7e and Supplementary Fig. 7c). Interestingly, cluster 1 is also enriched with Paneth cells gene, suggesting the T cell induction of Lgr5 cell differentiation into secretory lineages (Supplementary Fig. 7e). By scRNA-seq trajectory analysis, the cells in the cluster 1 showed more mature and differentiated cells than the cells in the cluster 11 and 23 (Fig. 7f,g), demonstrating that the cluster 1 contains a mixture of healing cells, T cells, ISCs and Paneth-like cells, reflecting the crypt cells that can play a crucial role in mucosal healing (Fig. 7o). Importantly, the cells in cluster 1 express less HLA and cell adhesion genes in DKI than in WT controls (Supplementary Fig. 7g,h), suggesting that depleting STAT5 tetramer can increase T cell interaction with Lgr5 ISCs to improve healing, possibly by promoting cytokine secretion, such as IL-17A and TGF-β, other than increasing TCR-MHC or E-cadherin^15,17^ (Fig. 7m and Supplementary Fig. 7j). Indeed, we should also have used dissociated crypt cells for scRNA-seq to determine the relationship between crypt stem cells and infiltrating T cells. However, because the number of crypt T cells may be lost during experimental procedures, resulting in very low cell numbers insufficient for scRNA-seq analysis. Importantly, since we are also unsure how TCRδ T cells infiltrate into the crypts from LP, we used the entire mucosal cell dissociation for scRNA-seq analysis. Nevertheless, these data suggest the necessity to precisely analyze the differentiated crypt T types and characterize the activated ISCs using both trajectory-based differential expression analysis and connectome analysis of cell communication at single-cell resolution^59,60^.

Noticeably, the tetrameric STAT5 can interact with the H3K27 methylase EZH2 to form H3K27-trimethylated repressive chromatin^37^ and may directly regulate TCRγδ gene rearrangement^38^. *Mt1* is highly expressed in the intestinal antigen-presenting cells that can induce naive T cells to differentiate into T_reg_ cells by reducing the expression of MHC-II and CD86 while increasing IL-10 production^61,62^. Intriguingly, inhibition of *Stat5* and *Stat3* decreases *Mt1* promoter binding, suggesting a requirement for STAT3 and/or STAT5 to bind the *Mt1* promoter^63^. Our data show that depleting STAT5 tetramers increased STAT5 binding to the *Mt1* promoter. Interestingly, STAT5 tetramer depletion did not significantly alter H3K4Me3 (Supplementary Fig. 8), suggesting that STAT5 tetramer results in *Mt1* repression of crypt T cell differentiation, possibly by forming a suppressive chromatin induced by H3K27Me3 markers. Taken together, these data indicate that tetrameric STAT5 depletion reduces STAT5 DNA binding, thereby releasing STAT5 repression of *Mt1*, leading to TCRγδ T cells retained and differentiated in the crypts. Furthermore, sc-ATAC-seq could be the next step to determine the chromatin accessibility of TCRγδ T cell transcription factors regulated by STAT5 at single-cell resolution^64^.

Human IBD–UC is characterized by increased numbers and activation of immune cells, including T cells, neutrophils, macrophages and dendritic cells; increased levels of T_H_1 and T_H_17 cytokines; and the presence of crypt hyperplasia and cryptitis. These lesions may be self-healing or develop as pseudopolyps over time, possibly through stem-like T cells^65,66^. Our data show significantly increased infiltration of CD3^+^TCRγδ T cells within the +4 ISC zone in IBD–UC but not in IBD–Crohn’s disease (Fig. 1a–d and Supplementary Fig. 1e,f). Thus, we ask whether these T cells are protective in colonic ISC regeneration. Our data revealed that these increased TCRγδ T cells mainly reside in inflamed colonic crypts, and some are cytoplasmic STAT5-positive, suggesting STAT5 dimeric or tetrameric activation (Fig. 1d,f). Interestingly, bulk RNA-seq showed that patients with UC with higher CD3^+^CD4^−^CD8^+^TCRγδ^+^pYSTAT5^+^ in blood showed increased IL-2, IL-15, TCR and progenitor regeneration signatures, whereas reduced stemness and pluripotency (Fig. 1g–j), suggesting that these patients with IBD–UC have a greater capacity for epithelial repair. However, a further study should be conducted with a bigger cohort of patients with IBD–UC or Crohn’s disease. Human scRNA- or TCR-seq analysis may increase analytical efficiency compared with current RNA-seq, IH and FACS analyses.

Our study identified a new type of crypt TCRγδ T cells that can protect against IR- and colitis-induced injury during ISC regeneration. Our research not only identifies a type of immune niche cell that can be targeted to preserve ISCs but also suggests a cell therapy to improve IBD healing.

### The paper explained

**Problem**

It is largely unexplored how ISCs are regulated during inflammation, leading to a lack of healing medicines for IBD. Intra-epithelial T lymphocytes regulate IEC repair and influx into crypts during IBD (UC). However, whether T lymphocytes can protect ISCs is unclear. STAT5 can form dimers and tetramers that are critical for T cell maturation or ISC regeneration, yet it is not known what the function of tetrameric STAT5 is during immune-mediated gastrointestinal regenerative repair.

**Results**

Our study shows increased numbers of crypt STAT5^+^TCRγδ^+^ T cells in patients with IBD with UC compared with healthy patients. Intra-crypt TCRγδ T cells can form an immune niche for ISC regeneration. STAT5 dimerization, at the expense of tetramers, leads to TCRγδ T cell niche formation. STAT5 tetramer depletion decreases STAT5 binding on the *Metallothionein 1* (*Mt1*) locus to release *Mt1* repression and induces crypt TCRδ^+^ T cells to secrete IL-17A that enhances ISC regeneration.

**Impact**

Our data demonstrate intra-crypt TCRγδ T cells as a new type of immune niche cell. The tetrameric STAT5 suppresses the formation of the crypt T cell niche. Interrupting STAT5 tetramers promotes the expansion of crypt TCRγδ cells, providing a target for regenerative repair of ISCs during IBD–UC.

## Supplementary information

Supplementary Information
