# Supplementary material for: Tetrameric STAT5 regulates the formation of immune niche cells to protect stem cell regenerative repair against mucosal inflammation
Source: Exp Mol Med. 2026 May 1;58(5):1495–509. doi: 10.1038/s12276-026-01716-0 (PMC13234138; doi:10.1038/s12276-026-01716-0)
Supplement: Supplementary file 1 — Supplementary Information [file 12276_2026_1716_MOESM1_ESM.pdf]

Tetrameric STAT5 Regulates the Formation of Immune Niche Cells to Protect Stem Cell Regenerative Repair Against Mucosal Inflammation

Running title: Regulation of Crypt Niche Cell Formation

Haifeng Li [2], Xueli Ding [1,3], Sabrina Fabec [1], Ahmed Bakheet [1], Wen Gao [1], Mingquan Song [3], Ruixue Liu [2], Xiaonan Han [1,4#]

[1] Division of Hematology and Oncology, and Division of Cancer Biology, Department of Medicine, The MetroHealth System (MHS), Case Western Reserve University (CWRU) School of Medicine, Cleveland, OH, USA. [2] Institute of Laboratory Animal Sciences (ILAS), Peking Union Medical College (PUMC), Beijing 100021, China. [3] Department of Gastroenterology, the Affiliated Hospital of Qingdao University, Qingdao, Shandong 266003 or 266005, China. [4] Cancer Genomics and Epigenomics Program, Case Comprehensive Cancer Center (CCCC), Case Western Reserve University (CWRU), Cleveland, OH, USA

Supplemental Materials and Methods

Materials. Unless otherwise noted, all chemicals and antibodies were purchased from Sigma-Aldrich (St. Louis, MO). Antibody specific for CD3 (Cat #78588, #99940 and #85061), EZH2 (Cat #5246), H3K4Me3 (Cat #9751), H3K27Me3 (Cat #9733), TCRγδ (Cat # 55750), STAT5 (Cat #94205) and tyrosine phosphorylation–specific STAT5 antibody (Tyr642/699, Cat #9359) were purchased from Cell Signaling Technology (CST, Danvers, MA USA). Antibodies specific for GFP (Cat #ab13970), LGR5 (Cat #ab75850), and Ki67 (Cat #16667) were purchased from Abcam (Cambridge, MA, USA). Antibodies specific for CD4 (Cat #550954) and CD8 (Cat #557654) were purchased from BD Pharmingen (San Diego, CA, USA). Antibodies specific for CD3 (Cat #100341) and TCRγδ (Cat #118101 and #118108) were purchased from BioLegend (San Diego, CA, USA). Antibodies specific for STAT5 (Cat #sc-74442) were from Santa Cruz (Dallas, TX, USA). For mouse organoid culture and maintenance, Matrigel was purchased from Corning (Bedford, MA, USA), and Noggin (Cat #1967-NG-025/CF), EGF (Cat #2028-EG-200), Wnt-3A (Cat #5036-WN-010/CF) and R-Spondin (Cat #3474-RS-050) were obtained from R&D Systems (Minneapolis, MN, USA). IntestiCult™ Organoid Growth Medium (mouse) was from STEMCELL Technologies (Vancouver, Canada, USA). Click-iT® EdU Cell Proliferation Kit for FACS and Imaging kit (Cat #06005 and #C10086, ThermoFishser Scientific, USA) and Annexin V conjugates apoptosis kit (Cat #A23204, ThermoFishser Scientific, USA and Cat #P04D11, Gene-Protein Link, Beijing, China). All materials used for Immunohistochemistry (IH), HRP-conjugated anti-rabbit/mouse IgG, and donkey serum were from Vector Laboratories (Newark, CA, USA). For immunofluorescence (IF), secondary Alexa Fluor ®488/594/647-conjugated donkey anti-mouse/rabbit/chicken IgG were purchased from Jackson ImmunoResearch

Laboratories (West Grove, PA, USA), and Zenon™ Rabbit IgG Labeling Kits were from Thermo Fisher Scientific (Waltham, MA, USA).

Biopsies and surgical specimens from IBD patients[1]**.**

Colonic biopsy specimens and blood samples from adult patients, including 20 Crohn’s disease (CD), 23 Ulcerative colitis (UC), and 10 healthy control patients, were obtained from the Department of Gastroenterology, the Affiliated Hospital of Qingdao University, Qingdao, Shandong, P.R. China. The details of patient samples are listed in **Supplementary Table 1**. Paraffin-embedded surgical specimens were from the division of pathology, MetroHealth Medical Center (MHMC), Cleveland, USA, and the pathology division, University of Cincinnati Medical Center, Cincinnati, USA.

Before processing for HE, histology, RNA extraction, and patient enrollment, IRB or de-identified IRB protocols for using IBD patient samples were approved, respectively, by the Institutional Review Board (IRB) protocols at MHMC/CWRU, Cleveland, OH, USA (XH, IRB21-00237), the ethics committee of Affiliated Hospital of Qingdao University, Qingdao, P.R. China (QYFY-WZLL-28569 and 29546), and de-identified Cincinnati Children’s Hospital Medical Center (CCHMC), Cincinnati, OH, USA (XH, IRB2009-1680). All research was performed in accordance with relevant guidelines/regulations using human samples.

Animal resources and maintenance[1]**.** The animal study protocols were approved by the Institutional Animal Care and Use Committee (IACUC) at MHMC and CWRU, Cleveland, OH, USA (IACUC 2020-0081) and at ILAS, Beijing, P. R. China (LHF21001). All mice were maintained in specific pathogen-free (SPF) conditions in the MHMC/CWRU and ILAS Animal Care Facility. All experiments were performed in accordance with the relevant guidelines and regulations for the use of live vertebrates. Inducible *Stat5* depletion (*Stat5*$^{-/-}$) and constitutively

active *Stat5a* mice (cS5a) and *Stat5a–Stat5b* double knock-in (DKI) N-domain mutant mice (DKI, hereafter called STAT5 tetramer deficient mice) were generated as previously reported[2, 3]. *Rosa26-cre*ERT2 (*RsCre*ER) mice were purchased from Jackson Lab (Strain #:008463, Bar Harbor, ME). Detailed procedures for crossing and genotyping *RsCre*ER;*stat5*$^{f/f}$ or *cS5*$^{f/f}$, and *Lgr5Cre*ER;DKI mice were shown in the **Supplemental Figures** (**Supplementary Fig. 1a**)**.** Genotyping Primers were listed in **Supplementary Table 2.**

Radiation-induced injury models[3]**.** Eight-week-old DKI and WT control mice were exposed to 13 Gy whole-body γ-radiation for 10 minutes at the ILAS or the WRU Comprehensive Cancer Center Core.[4]. The intestinal tissues were inspected for gross and histological abnormalities[4, 5]. Radiation Injury Scores (RIS) and mucosal ulceration of radiation-induced intestinal mucositis were determined as previously described[6]. RIS is a composite histopathologic scoring system that our laboratory has extensively improved. Briefly, Scores of γ-radiation-induced ileal mucosal damage (scores: 1, 2, and 3), crypt loss (scores: 1, 2, and 3), mucosal ulcerations (scores: 1, 2, and 3), and thickening of the intestinal wall (scores: 1, 2, and 3) were combined as RIS. Numbers of proliferating crypts or regenerated crypts ("microcolonies") were quantified as crypts per mm under a microscope (magnification 100 ×) and were confirmed by Ki67 Immunohistochemistry (IH).

Animal model of colitis[7, 8]**.** DKI and WT littermate control mice were orally given 2.5% dextran sulphate sodium (DSS) (m.w. 36,000–50,000; MP Biomedicals) in water 7 days following with 5-day water recovery specific for inflammation-induced colonic ISC repair or three cycles of 5-day DSS following 16-day water recovery specific for apoptosis-induced colonic ISC regeneration[3, 9]. Mice were sacrificed, and the colon was removed. Scoring parameters included quantification of the area of the middle and distal colon involved, edema, epithelial monolayer erosion/ulceration,

crypt loss/damage, and infiltration of immune cells into the mucosa. Total disease scores were the mean of all combined scores per genotype. Regenerated colonic crypts adjacent to ulcers were determined with Ki67 IH, and colonic ISC regeneration was expressed as the number of $Ki67^+$ IECs per regenerated crypt (magnification 100 ×).

IH, Immunofluorescence (IF), and Quantitative Real-time PCR (QPCR)[1, 10]**.** Anti-Ki67, Lysozyme (Lyso), Apoptosis Inhibitor 5 (API5), EdU, CD3, TCRγδ, STAT5, and pYSTAT5 antibodies listed in **Supplementary Table 3** were used to detect crypt ISC proliferation, Paneth cell hyperplasia, T cell and STAT5 activation. The *Lgr5Cre*ER and *Lgr5Cre*ER;DKI mice were intraperitoneally (i.p.) administered with EdU (Cat #A10044, ThermoFisher Scientific) and then euthanized three hours later. Anti-EdU, GFP, Ki67, or LGR5 antibodies listed in **Supplementary Table 3** were used to detect Lgr5 ISC self-renewal or intestinal epithelial cell (IEC) proliferation with EdU IF staining following the Click-iT® EdU Imaging Kit instructions. The mRNA levels of cytokines, ISC markers, and Metallothionein-1 (Mt1) were measured by qPCR, with the primers listed in **Supplementary Table 4**.

Briefly, IH was performed using 4 μm-thick formalin-fixed and paraffin-embedded sections or 10 μm-thick paraformaldehyde (PFA, Cat #158127, Sigma-Aldrich, St Louis, MO, USA)-fixed frozen sections for IF staining. The sections were steamed in Tris-EDTA buffer at pH 9.0 for IH. Endogenous peroxidase was blocked with 3% H2O2 in methanol for IH or 3% hydrogen peroxide in PBS for IF. 2.5% horse or goat serum (Vector Laboratories Inc., Newark, CA) to block non-specific binding. The sections were incubated with the primary antibody CD3 in a 1:100 dilution, GFP in a 1:200 dilution, Ki67 in a 1:200 dilution, LGR5 in a 1:50 dilution, TCRγδ in a 1:100 dilution, pYSTAT5 in a 1:50 dilution, and STAT5 in a 1:200 dilution at 4°C overnight. The slides were then incubated with HRP- or fluorescein-conjugated secondary antibodies for 45 min at room

temperature (RT). Lastly, slides were incubated with diaminobenzidine (DAB) (Vector Laboratories Inc.) at RT for 3 min to visualize IH under a Zeiss microscope (Axiocam 712, Zeiss Inc., Germany), or for imaging IF under a fluorescent confocal microscope (The Leica TCS SPE confocal, Leica Inc., Germany). Using a quantitated software from Vectra 3 automated quantitative pathology imaging system (PerkinElmer, Waltham, MA), the frequency of Lgr5 and/or EdU, Ki67, CD3 and/or TCRγδ and/or STAT5 IH or IF staining in the crypts of DKI, WT, healthy control mice or IBD-UC patients were quantified as average numbers per villus or +4 stem cell zone at crypt bases. The results are expressed as Mean ± SEM.

Murine total RNA was isolated from SI, Co tissues, or crypts using TRIzol (Thermo Fisher Scientific, Waltham, MA) and stored at -80 ºC. Cytokine, ISC, and Mt1 mRNA levels were measured with qPCR using SYBR™ Green master mixes and the primers shown in **Supplementary Table 4**.

Organoid's culture, IR treatment, IL-17A stimulation, EdU incorporation, and Medium transfer culture[7, 11]**.** Intestinal and colonic crypts were isolated from DKI and WT control mice or IR-treated DKI and WT, then dissociated with chelation buffer (1mM EDTA, 5 mM EGTA, 0.5 mM DTT, 43.3 mM sucrose, and 54.9 mM sorbitol). The crypts were filtered and resuspended in Matrigel containing 50 ng/ml EGF, 100 ng/ml Noggin, and 500 ng/ml R-spondin, with or without Wnt3a (100 ng/ml). Before any experiments, intestinal enteroids and colonic colonoids were recovered and passaged at least two generations. The main medium obtained from the initial culture of IR-treated mouse crypts was preserved for transferring to the passaged organoid culture. These organoids were *in vitro* differentiated from day 1 to day 14, then exposed to 2 Gy IR for 5 mins. 2 hrs after EdU incorporation (Click-iT® EdU Cell Proliferation Kit for FACS and Imaging kit), the organoids were fixed with 4% PFA, or total RNA was extracted with TRIzol. The live

organoids or the sections from OCT-embedded organoids were imaged and video-recorded with ImageXpress Micro Confocal High-Content Imaging System (Molecular Devices, San Jose, CA). Lgr5 ISC self-renewal or proliferation was imaged and quantified as the number of buds, multiplicity (org3^{+}, org2, org1, and Sphere), and size of spheres by the ImageXpress® system (Molecular Devices). Lgr5-GFP buds were counted in each well, and the number of buds produced by an individual enteroid or colonoid per day was expressed as budding curves. Organoid-forming capacity in the presence or absence of IR was determined by counting final organoid survival and initial-grown organoids to calculate the percentage of regenerated organoids. Six replicate wells per mouse were assessed in each experiment.

The enteroids were derived from intact Lgr5 crypts dissociated from Lgr5CreER mice. The enteroids were stimulated with IL-17A (1, 10, or 50 ng/ml) for 6 days. GFP or GFP+DIC images of enteroids or IL-17-treated samples were recorded from Day 1 to Day 5. Lgr5 colony formation or colony growth in the 1 ng/ml, 20 ng/ml, or 50 ng/ml-treated enteroids, as well as budding curves of enteroids treated with 1, 10, or 50 ng/ml, were measured from 100 to 200 Lgr5^{+} or Lgr5^{-} intact crypt-derived enteroids. The enteroids harvested on Day 5 were processed for total RNA extraction. The expression levels of Lgr5, Atoh1, Lyz1, Olfm4, and Ascl2 were quantified using qPCR. The enteroids treated with 1, 10, or 50 ng/ml were incubated with EdU on Day 5, then harvested and fixed with paraformaldehyde (PFA). Sections of 10 μm thickness were stained with anti-GFP and EdU IF. Lgr5^{+}, EdU^{+}, or Lgr5^{+}EdU^{+} organoid cells were quantified as the number per organoid crypt. Six replicate wells per mouse were assessed in each experiment.

Flow Cytometry (FACS) analysis[3, 10]**.** Peripheral blood mononuclear cells (PBMCs) were collected from IBD-UC patients (**Supplementary Fig. 1a,b**). Mouse SI crypts, mesenteric lymph nodes (MLNs), and spleen were dissociated into single cells (**Supplementary Fig. 2a**).

Lgr5 FACS analysis was performed using isolated SI epithelia from *Lgr5Cre*ER and *Lgr5Cre*ER;DKI mice after i.p. administration of EdU at 50 mg/kg. Crypt IECs were extracted with 2 mM EDTA and manual shaking, then filtered through a cell strainer to generate a single-cell suspension. Singlet discrimination was sequentially performed using plots for FSC (FSC-A vs. FSC-H) and SSC (SSC-W vs. SSC-H). Scatter characteristics and 7-AAD staining were used to exclude dead cells. Lgr5^{+} ISCs were identified by their endogenous GFP expression and EdU labeling. Intra-IEC or crypt T cells were stained with anti-mouse CD3, CD4, CD8, and TCRγδ antibodies. Human PBMCs were stained with anti-human CD3, CD4, CD8, CD45, TCRαβ, or TCRγδ antibodies (**Supplementary Fig. 1a** and **Supplementary Table 3**). All FACS experiments were performed on the BD LSRFortessa™ Cell Analyzer (BD Biosciences), and FACS data were analyzed using FlowJo™ software (Tree Star, OR).

RNA-Sequencing (Seq) analysis[10]**.** Human colonic tissue is frozen in liquid nitrogen. The mice were euthanized after IR treatment, and the small intestines (SI) were isolated, organoids were differentiated, and the organoids were frozen in liquid nitrogen for RNA-seq. Total RNA was extracted from SI, and the concentration and quality were assessed using a Nanodrop 2000 (Agilent Technologies, Santa Clara, CA, USA). Magnetic beads with Oligo (dT) were used to purify total RNA according to the manufacturer's instructions (Thermo Fisher Scientific, Waltham, MA, USA). The fragments were generated using a fragmentation buffer (Thermo Fisher Scientific, Waltham, MA, USA), then added to the reverse transcriptase reaction to generate cDNA. The adaptor was used to make the sticky end and to convert it into the flat end. Illumina Novaseq 6000 (Illumina, San Diego, CA, USA) was used for sequencing, with a read length of 2 × 150 bp. The data were analyzed using the free online platform of the Majorbio Cloud (www.majorbio.com). The raw data were submitted to the NCBI Sequence Read Archive (SRA). Bioproject numbers are

PRJNA1200789 for human colonic tissues, PRJNA1423895, PRJNA845965, and PRJNA705806 for mouse tissues, and PRJNA721570 for organoids.

Chromatin Immunoprecipitation (ChIP) QPCR and Sequencing (ChIP-seq). The SI crypts from DKI and littermate control mice were used to perform ChIP using the simple-ChIP kit according to the manufacturer's protocol (CST and New England Biolabs (NEB), Ipswich, MA, USA). PFA achieved covalent fixation of the protein-DNA complexes. Chromatin was digested with micrococcal nuclease (Thermo Fisher Scientific, Waltham, MA, USA) and Q800 Covaris® Ultrasonicator (Covaris, Woburn, MA, USA) into 150-900 bp DNA fragments. STAT5 (A-9, sc-7442, Santa Cruz, Dallas, TX, USA) or STAT5 (Cat #94205), H3K4Me3 (Cat #9751), and H3K27Me3 (Cat #9733) antibodies (CST) were used to immunoprecipitate the sheared chromatin complex that was prebound to magnetic protein G (Cat #70024, CST). Ideally, the starting weight for a single ChIP is 25 mg of tissue or 5×10^{7} cultured cells. 1 ml PBS solution with protease inhibitors (Cat #9005S, CST) was added to 25 mg SI crypts, then supplemented with 90 µL of fresh 18.5% PFA and shaken at room temperature for 20 mins. 100 µL of 10 × Glycine solution was added to terminate the cross-linking. The supernatant was discarded after centrifuging at 500g at 4 °C for 5 mins. The pellets were repeatedly washed with 1 mL of PBS containing the protease inhibitor. The products were lysed and stored at −80 °C.

25 mg cross-linking products were resuspended in 1 ml 1× buffer A (250 µl 4× buffer A (Cat #7006, CST) + 750 µl H_2O + 0.5 µl 1M DTT + 5 µl 200 × Protease inhibitors). ChIP analyses are performed with Cell Signaling Technology (Cat #9005s, CST). DNA was purified by the MinElute PCR Purification Kit (Cat #28006, QIAGEN, NRW, Germany). *Mt1* qPCR was performed with the immunoprecipitated DNA using the primers specific for GAS binding motif (TTCTCGAA) on

the *Mt1* gene (Forward, 5’-AAGAGTCAGTTGGGACACCTT-3’; reverse, 5’-CGAGACAATACAATGGCCTCC-3’)[3].

According to the manufacturer's instructions, the ChIP-Seq library was prepared using original Ultra II DNA Library Kits (Cat #E7645, NEB). Briefly, the extracted DNA was ligated to specific adaptors, followed by deep sequencing in the Illumina Novaseq 6000 using 150 bp paired-end. Raw data (raw reads) of fastq format were first processed through in-house Perl scripts. In this step, clean data (clean reads) were obtained by removing reads containing adapters, poly-N, and low-quality reads from the raw data. The clean reads were mapped to the reference genome using Bowtie2. Those high-quality mapping reads were subjected to further peak calling. Macs2 was used to call peaks with q-values < 0.05. The HOMER's find Motifs Genome.pl tool was used for Motif analysis. Peaks were annotated by the ChIPseeker package. Bioproject numbers are PRJNA1197577 and PRJNA748855.

Single cell (sc)-RNA-seq. Mice were executed, and the intestines were collected, flushed with cold Dulbecco's Phosphate-Buffered Saline (DPBS, Cat #C14190500BT, Gibco, Carlsbad, CA, USA). The intestinal mucosa were inverted using a catheter, chopped into small pieces (about 1×1×1 mm3), then transferred to centrifuge tubes, with an appropriate amount of Dispase (Cat #07913, STEMCELL Technologies, Vancouver, BC, Canada) added, and shaken at a specific temperature for 30 mins. Following centrifugation, the supernatant was decanted and discarded. The resuspended cell pellets were resuspended in red blood cell lysis buffer and incubated for 3 minutes at room temperature, then Centrifuged (100 g, 4 °C, 3 min). The samples were lastly resuspended in PBS. Then, the cell suspensions (300-600 living cells per ml determined by Count Star) were loaded on a Chromium Single Cell Controller (10×Genomics) to generate single-cell gel beads in emulsion (GEMs) by using Single Cell 3’ Library and Gel Bead Kit V3 (10×Genomics,

Cat #120237, 10×Genomics, San Francisco, SFO, USA) and Chromium Single Cell A Chip Kit (10×Genomics, Cat #120236, 10×Genomics) according to the manufacturer's protocol. Roughly 10,000 cells were added to each channel, with a targeted cell recovery estimate of 7,500. The captured cells were lysed, and the released RNA was barcoded through reverse transcription in individual GEMs[12]. GEMs were reverse transcribed in a C100 Touch Thermal Cycler (Bio-Rad, Hercules, CA, USA) by programming at 53 °C for 45 min, 85 °C for 5 min, and holding at 4 °C. After reverse transcription, single-cell droplets were broken. The single-strand cDNA was isolated and cleaned with Cleanup Mix containing DynaBeads (Thermo Fisher Scientific). cDNA was generated and amplified, and the quality was assessed using the Agilent 4200. Single-cell RNA-seq libraries were prepared using Single Cell 3' Library Gel Bead Kit V3 following the manufacturer's instructions. Sequencing was performed on an Illumina Novaseq 6000 with a sequencing depth of at least 100,000 reads per cell and a pair-end 150 bp (PE150) read length.

Raw FASTQ files were mapped to the Reference genome (Mouse) using Cell Ranger 3.0 (10× Genomics). To create Cell Ranger-compatible reference genomes, the references were rebuilt according to instructions from 10× Genomics (https://support.10xgenomics.com/single-cell-geneexpression/software/pipelines/latest/advanced/ references), which performed alignment, filtering, barcode counting, and UMI counting. Digital gene expression (DGE) matrices were generated for all samples following alignment. Gene expression analysis and cell type identification were analyzed using the Seurat V2.0 pipeline (http://satijalab.org/seurat/) after filtering and normalization, another R toolkit for single-cell transcriptomics[13]. For a gene to be differentially expressed in a cluster, it must have a log-fold change greater than 0.25 and reach the statistical significance of an adjusted $p<0.05$ as determined by the Wilcox test. The specific data preprocessing steps are listed as follows.

Cell Type Annotation Cell type was annotated by singleR (https://bioconductor.org/packages/devel/bioc/html/SingleR.html). Performs unbiased cell type recognition from single-cell RNA sequencing data by leveraging reference transcriptomic datasets of pure cell types to infer the cell of origin of every single cell independently. Finally, the cell clusters were annotated to the known biological cell types using canonical markers and published markers (**Supplementary Table 5**)[14, 15].

Cellranger pipeline. The Cell Ranger software was obtained from the 10× Genomics website at https://support.10xgenomics.com/single-cell-gene-expression/software/downloads/latest. Alignment, filtering, barcode counting, and UMI counting were performed with the cellranger count module to generate a feature-barcode matrix and determine clusters. Dimensionality reduction was performed using PCA, and the first 10 principal components were used to generate clusters using the K-means and graph-based algorithms, respectively.

Seurat pipeline. The other clustering method is Seurat 3.0(R package). Cells whose gene number was less than 200, or gene number ranked in the top 1%, or mitochondrial gene ratio was more than 25% were regarded as abnormal and filtered out. Dimensionality reduction was performed using PCA, and visualization was realized by TSNE and UMAP.

Enrichment Analysis. GO enrichment, KEGG enrichment, Reactome enrichment, and Disease enrichment (human only) of the cluster markers were performed using KOBAS software with the Benjamini-Hochberg multiple-testing adjustment, using the top 20 gene markers of the cluster. The results were visualized using the R package.

GSEA assay. GSEA was performed by using GSEA software version 2.2.2.4, which uses predefined gene sets from the Molecular Signatures Database (MSigDB v6.2). All genes detected in all cells of one sample were used. Gene expression data were calculated as the mean UMI count

for a gene across one cluster and the remaining clusters, respectively. The minimum and maximum criteria for selection of gene sets from the collection were 0 and 500 genes, respectively. GSEA analysis was used to compare the gene expression enrichment between WT and DKI.

Trajectory analysis. Single-cell trajectories were built with Monocle (R package), which introduced pseudotime. Genes were filtered by the following criteria: Expressed in more than 10 cells; The average expression value was greater than 0.1; Qval was less than 0.01 in different analyses. Trajectory analysis was used to analyze the lineage differentiation and cell maturation.

Above analyses were done in CapitalBio Technology (Beijing, China). The raw data were submitted to the NCBI Sequence Read Archive (SRA) under Bioproject number PRJNA1180729.

Transcript profiling. PRJNA845965, PRJNA705806, PRJNA721570 and PRJNA1200789 for RNA-seq, PRJNA1197577 and PRJNA748855 for ChIP-seq, and PRJNA1180729 for scRNA-seq.

Statistical Analysis. All data presented in the organoid culture are representative of at least 3 repeated experiments. The number of all vertebrate animals used in the experiments is greater than 5, with a mixed gender, unless stated otherwise. All data presented in Mean values with SEM were used for independent 2-tailed Student's t tests or 1-way ANOVA. All data compilation was done using the statistics software GraphPad Prism (7.0). *P* values of 0.05 or less were considered significant when using T-tests and analysis of variance (ANOVA).

					Serology examinations before starting biologic therapy					
**Diagnosis**	**Gender**	**Starting age**	**Age**	**BMI**	**WBC**	**HGB**	**PLT**	**Alb**	**ESR**	**CRP**
Con	M	NA	48	23.10	7.97	152.00	304.00	44.10	N/A	2.58
Con	F	NA	67	24.70	4.10	149.00	187.00	45.90	N/A	0.35
Con	F	NA	70	20.20	5.94	139.00	255.00	45.00	N/A	1.82
Con	F	NA	58	27.90	6.50	137.00	295.00	46.70	N/A	2.70
Con	M	NA	47	26.40	6.01	138.00	248.00	45.10	N/A	2.19
Con	F	NA	47	23.20	4.51	131.00	215.00	45.10	N/A	0.51
Con	M	NA	45	30.10	6.92	138.00	219.00	38.40	N/A	1.46
Con	F	NA	45	23.30	3.47	123.00	204.00	48.00	N/A	3.73
Con	M	NA	55	19.80	5.65	158.00	252.00	46.50	N/A	1.62
Con	M	NA	58	31.20	5.80	154.00	306.00	45.60	N/A	3.11
UC	M	45	75	20.07	13.76	88.00	491.00	24.30	79	63.47
UC	M	63	70	22.20	3.26	121.00	221.00	33.90	9	10.25
UC	M	70	70	17.30	7.66	80.00	335.00	23.30	51	133.44
UC	F	71	73	27.10	3.20	96.00	203.00	27.90	49	37.94
UC	M	44	64	26.03	7.36	127.00	278.00	33.42	23	49.87
UC	F	68	69	20.20	5.32	116.00	279.00	39.50	14	7.08
UC	M	54	64	26.40	8.08	140.00	279.00	39.00	10	8.55
UC	M	60	67	22.59	14.07	97.00	280.00	26.76	29	45.48
UC	M	62	65	24.80	15.06	101.00	308.00	32.15	60	35.06
UC	M	62	65	21.22	7.45	75.00	334.00	33.74	20	8.68
UC	M	72	78	23.90	12.16	87.00	256.00	36.90	50	11.69
UC	F	43	69	24.60	9.57	103	274.00	34.60	33	4.72
UC	F	72	72	21.50	6.84	105.00	350.00	32.40	88	128.52
UC	M	72	72	24.50	13.01	114.00	245.00	33.70	12	7.07
UC	M	54	68	19.10	4.81	75.00	31.00	29.60	7	14.85
UC	M	64	68	20.30	5.02	130.00	247.00	40.80	14	8.26
UC	M	75	76	24.20	6.33	116.00	452.00	30.90	40	3.34
UC	M	63	63	20.42	10.05	136.00	262.00	37.80	3	1.42
UC	M	64	64	22.72	5.39	100.00	462.00	33.06	70	1.50
UC	M	72	72	24.09	8.12	98.00	222.00	27.06	37	16.14
UC	M	64	66	17.30	6.00	136.00	180.00	41.36	10	1.43
UC	M	61	66	17.18	6.55	87.00	322.00	38.40	16	6.59
UC	M	62	62	20.20	6.05	84.00	432.00	27.80	31	31.57
UC	F	59	61	18.03	4.66	94.00	217.00	30.70	40	9.27
CD	M	5	54	24.86	0.99	6.72	158.00	7.00	38.25	280.00
CD	F	56	59	14.28	45.48	8.52	99.00	60.00	31.98	191.00
CD	M	26	27	17.37	15.29	4.81	130.00	16.00	36.9	336.00
CD	F	34	36	14.79	1.51	5.98	81.00	9.00	37.4	349.00
CD	M	33	43	23.18	0.45	4.75	161.00	2.00	40.87	181.00

CD	F	50	59	20.69	2.46	6.84	113.00	17.00	35.9	221.00
CD	F	30	42	24.54	3.50	5.24	120.00	16.00	38.8	274.00
CD	M	15	18	17.72	1.39	6.25	143.00	3.00	45	302.00
CD	M	58	59	22.96	1.64	5.00	145.00	2.00	38.4	154.00
CD	F	37	47	22.66	0.59	3.00	102.00	12.00	40.8	181.00
CD	M	34	39	17.92	0.34	7.96	140.00	7.00	43.95	203.00
CD	M	27	32	14.63	0.62	4.65	137.00	2.00	44.1	195.00
CD	M	22	26	22.10	2.96	7.05	137.00	3.00	41.7	306.00
CD	F	22	24	17.99	1.13	3.77	103.00	6.00	39.7	191.00
CD	M	34	25	18.52	23.58	10.71	121.00	15.00	33.3	291.00
CD	M	31	34	15.57	38.02	8.95	124.00	24.00	42.66	211.00
CD	F	55	56	21.48	54.30	6.03	116.00	51.00	34.9	270.00
CD	F	66	70	19.90	3.00	113.00	208.00	43.16	10	2.00
CD	M	68	73	24.40	4.73	87.00	229.00	39.05	27	3.29
CD	F	57	67	29.07	6.56	124.00	235.00	40.76	14	5.56
CD	F	66	69	21.48	3.52	117.00	229.00	39.43	7	38.81

Supplementary Table 1. The patient details of blood samples and biospecimens.

Gene name	Primer sequence (5'-3')	References
cS5f Forward	AGGCGACCATCATCAGCGAGC	[3]
cS5f Reverse	GAATGGAGAAATCTCGCGTCG	
Lgr5 common	CTGCTCTCTGCTCCCAGTCT	[3]
Lgr5 wild type	ATACCCCATCCCTTTTGAGC	
Lgr5 mutation	GAACTTCAGGGTCAGCTTGC	
DKI1	AAGGGACAGGAAGAGAGAAGG	[2]
DKI2	CCCATACAACACTTGCATCT	
DKI3	GCAAAACCACACGCTCGAC	
Rs26CreER-tdTomato 1	AAGGGAGCTGCAGTGGAGTA	[10]
*Rs26Cre*ER-tdTomato 2	CCGAAAATCTGTGGGAAGTC	
*Rs26Cre*ER-tdTomato 3	GGCATTAAAGCAGCGTATCC	
*Rs26Cre*ER-tdTomato 4	CTGTTCCTGTACGGCATGG	
Stat5f 1	GAAAGCATGAAAGGGTTGGAG	[7]
Stat5f 2	AGCAGCAACCAGAGGACTAC	
Stat5f 3	AAGTTATCTCGAGTTAGTCAGG	

Supplementary Table 2. Mouse genotyping primer sequences.

	Company	Cat #	Dilution
For **IHC/IF**			
CD3ε（D4V8L) Rabbit mAb	CST	99940	1:100
TCR γ/δ Ab	Santa Cruz	sc-19608	1:100
Stat5(D206Y) Rabbit mAb	CST	94205	1:200
Phospho-Stat5(Tyr694) (D47E7) XP Rabbit mAb	CST	4322	1:100
Rabbit monoclonal anti-Ki67	Abcam	ab16667	1:200
Rabbit monoclonal anti-API5	Abcam	ab323714	1:200
For **FACS**			
FITC anti-human TCRγδ	BD Biosciences	BC IM15171U	
PE anti-human TCRαβ	BD Biosciences	BC A39499	
PerCP-Fluor anti-human CD3	BD Biosciences	BD 340663	
Fluor V450 anti-human CD45	BD Biosciences	BD 3406548	
APC-H7 anti-human CD4	BD Biosciences	BD 560158	
APC-R700 anti-human CD8	BD Biosciences	BD 565192	
Alexa 488 anti-human pYSTAT5	BD Biosciences	BD 612598	
PerCP-Cy7 anti-mouse CD8a	BD Pharmingen	557654	
PerCP-Cy5.5 anti-mouse CD4	BD Pharmingen	550954	
Brilliant Violet 421 anti-mouse CD3ε	Biolegend	100341	
PE anti-mouse TCRγ/δ	BioLegend	118108	
APC anti-mouse CD25	BD Pharmingen	557192	
PE anti-mouse FOXP3	BD Pharmingen	560414	
BV605 anti-mouse IL-17A	Biolegend	506927	
PE-Cy7 anti-mouse pYSTAT5	Biolegend	936907	
APC-Cy7 anti-mouse CD3ε	Biolegend	100221	
PerCP-Cy5.5 anti-mouse CD4	Biolegend	100539	
Bv510 anti-mouse CD8a	Biolegend	100751	
Annexin V-Alexa Fluor 647/PI Kit	Gene-Protein Link	P04D11	
EdU	Click-iT EdU-647 cell proliferation kit Gene-Protein Link	P04D45	
For **ChIP**			
Anti-mouse STAT5	Santa Cruz	7442	
Anti-mouse STAT5	CST	94205	
Anti-mouse H3K4Me3	CST	9751	
Anti-mouse H3K27Me3	CST	9733	

Supplementary Table 3. Antibody list.

Gene name	Primer sequence (5’-3’)	References
GAPDH Forward	GGTGGGTGGTCCAAGGTTTC	[3]
GAPDH Reverse	TGGTTTGACAATGAATACGGCTAC	
IL-4 Forward	GGCTTTTCGATGCCTGGATT	[16]
IL-4 Reverse	TTTGCATGATGCTCTTTAGGCTTT	
IL-6 Forward	CAAAGCCAGAGTCCTTCAGAGAGATAC	[7]
IL-6 Reverse	GGATGGTCTTGGTCCTTAGCCAC	
IL-7 Forward	GGTCATCATGACTACGCCCG	[17]
IL-7 Reverse	GGAGGATGCAGCTAAAGTTCG	
IL-10 Forward	TGGTTTCTCTTCCCAAGACC	[7]
IL-10 Reverse	CCCTTTGCTATGGTGTCCTT	
IL-12(p35) Forward	AAGACATCACACGGGACCAAA	[18]
IL-12(p35) Reverse	CAGGCAACTCTCGTTCTTGTGTA	
IL-13 Forward	AGGAGCTGAGCAACATCACAC	[19]
Il-13 Reserve	CCATAGCGGAAAAGTTGCTT	
Il-15 Forward	GTTGACGAGCAATGAGACGAT	[20]
Il-15 Reverse	GCCACACTTCTCTTTTTGTCC	
IL-17 Forward	TTTAACTCCCTTGGCGCAAAA	[7]
IL-17 Reverse	CTTTCCCTCCGCATTGACAC	
Il27 (*p28*) Forward	GGCTATGTCCACAGCTTTGCT	[18]
Il27 (*p28)* Reverse	CGAAGTGTGGTAGCGAGGAA	
IL-23 Forward	CATGGGGCTATCAGGGAGTA	[7]
IL-23 Reverse	GACCCACAAGGACTCAAGGA	
Mt1 Forward	TCACCAGATCTCGGAATGG	[21]
Mt1 Reverse	AAGAACCGGAATGAATCGC	
Mt2 Forward	CGCGCTCACTGACTGCCTTC	[21]
Mt2 Reverse	CTGGGAGCACTTCGCACAGC	
Lgr5 Forward	CGGAGGAAGCGCTACAGAAT	[22]
Lgr5 Reverse	CTGGGTGGCACGTAGCTGAT	
Ascl2 Forward	AAGCACACCTTGACTGGTACG	[23]
Ascl2 Reverse	AAGTGGACGTTTGCACCTTCA	
Olfm4 Forward	CAGCTGCCTGGTTGCCTCCG	[24]
Olfm4 Reverse	GGCAGGTCCCATGGCTGTCC	
Atoh1 Forward	GTAAGGAGAAGCGGCTGTG	
Atoh1 Reverse	AGCCAAGCTCGTCCACTA	
Lyz1 Forward	TGACATCACTGCAGCCATAC	[25]
Lyz1 Reverse	TGGGACAGATCTCGGTTTTG	

Supplementary Table 4. Primer sequence list.

**Stem Cells**	**Normal stem cells**	Mki67	Lgr5	Olfm4	Bmi1	Cd24a	Cd44	Ascl2	Uri1	Mex3a
		Hopx	Sox9	Krt19	Lrig1	Ccl25	Akt1	Akt2	Ptger4	Axin2
**Epithelial cells**	**Enterocytes**	Vil1	Fabp6	Cftr	Adh1	Apopa1	Rik	Aspa	Fabp1	
		Krt20	Reg4	Rps2	Apoc3	Alpi	Cnnb1	Reg1	Slc7a9	Clec2h
	**TA cells**	Mki67	Apoc3	Rps25	Gpd1	Alpi	Rps10	Eef1b2	Fabp1	Dpp4
	**Enteroendocrine cells**	Chgb	Neurog3	Cyp2c29	Slc5a1	Sox4	Anxa6	Olfm1		
	**Tuft cells**	Lrmp	Dclk1	Rgs13	Avil	Gale	Eppk1	Dclk1	Adgrg2	
	**Paneth cells**	Defb1	Lyz1	Reg4	Cd117	CD44	Cd24	Sox9	Fzd9	Axin2
	**Goblet cells**	Muc2	Fcgbp	Ccl9	Tpsg1	Tnfrsf21	Itga2	Spdef	Reg4	
**Stromal cells**	**Telocytes**	Pdgfra	Pdgfrb	Acta2	Gli1	Foxl1				
	**Myofibroblasts**	Acta2	Vim	Palld	Actg2					
	**Endothelial cells**	Pecam1	Ly6a	ly6c1	Cd34	Cdh5	Pecam1			
	**Gial cells**	Ncam1	Cdh2	Vim	Sox9					
	**Lymphatic Vessel**	Lyve1	Flt4							
**T/B Lymphocyte**	**CD8 T cells**	Cd3e	Cd8a	Cd8b	Bcl6	Cd27	Tcf7	Id2		
	**CD4 T Cells**	Cd3e	Cd4	Cd3g	Cd3d					
	**B Cells**	Ebf1	Cd19	CD20	Ighm	Ighd				
	**Circulating Monocyte**	Ly6c2								
**Myeloid**	**Dendritic cells**	Itgae	Itgax	H2-Ab1	Cd209a					
	**Granulocytes**	Ly6g								
	**Recruited Monocytes**	Ly6c2	Ccr2	Itgam	H2-Ab1	Ccl2	Cd68	Adgre1		
	**Macrophages**	Lyz1	Itgax	Cd68	H2-Ab1	Csf1r	Itgam	IL10	Itgax	

Supplementary Table 5. Cell marker annotation in scRNAseq.

References

1. Zhang, D. *et al.* Monogenic deficiency in murine intestinal Cdc42 leads to mucosal inflammation that induces crypt dysplasia. *Genes Dis.* **11**, 413–429 (2024).
2. Lin, J. X. *et al.* Critical Role of STAT5 transcription factor tetramerization for cytokine responses and normal immune function. *Immunity* **36**, 586–599 (2012).
3. Gilbert, S. *et al.* Activated STAT5 Confers Resistance to Intestinal Injury by Increasing Intestinal Stem Cell Proliferation and Regeneration. *Stem Cell Reports* **4**, 209–225 (2015).
4. Hua, G. *et al.* Crypt base columnar stem cells in small intestines of mice are radioresistant. *Gastroenterology* **143**, 1266–1276 (2012).
5. Han, X. *et al.* Loss of GM-CSF signalling in non-haematopoietic cells increases NSAID ileal injury. *Gut* **59**, 1066–1078 (2010).
6. Akpolat, M., Kanter, M. & Uzal, M. C. Protective effects of curcumin against gamma radiation-induced ileal mucosal damage. *Arch. Toxicol.* **83**, 609–617 (2009).
7. Gilbert, S. *et al.* Enterocyte STAT5 promotes mucosal wound healing via suppression of myosin light chain kinase-mediated loss of barrier function and inflammation. *EMBO Mol. Med.* **4**, 109–124 (2012).
8. Sun, W. *et al.* Protocol for colitis-associated colorectal cancer murine model induced by AOM and DSS. *STAR Protoc.* **4**, 102105 (2023).
9. Greten, F. R. *et al.* IKKbeta links inflammation and tumorigenesis in a mouse model of colitis-associated cancer. *Cell* **118**, 285–296 (2004).
10. Liu, R. *et al.* Constitutive STAT5 activation regulates Paneth and Paneth-like cells to control Clostridium difficile colitis. *Life Sci. Alliance* **2**, e201900310 (2019).
11. Gao, W. *et al.* Generating vasculature and immune cell-chimeric tumoroids via intraperitoneal Xenograft. *Cancer Lett.* **638**, 218136 (2026).
12. Zheng, G. X. *et al.* Massively parallel digital transcriptional profiling of single cells. *Nat. Commun.* **8**, 14049 (2017).
13. Butler, A., Hoffman, P., Smibert, P. & Satija, R. Integrating single-cell transcriptomic data across different conditions, technologies, and species. *Nat. Biotechnol.* **36**, 411–420 (2018).
14. He, X. *et al.* Tumor-initiating stem cell shapes its microenvironment into an immunosuppressive barrier and pro-tumorigenic niche. *Cell Rep.* **36**, 109674 (2021).
15. Biton, M. *et al.* T Helper Cell Cytokines Modulate Intestinal Stem Cell Renewal and Differentiation. *Cell* **175**, 1307–1320 (2018).
16. Bentley, J. K. *et al.* Periostin is required for maximal airways inflammation and hyperresponsiveness in mice. *J. Allergy Clin. Immunol.* **134**, 1433–1442 (2014).
17. Puel, A. *et al.* Defective IL7R expression in T(-)B(+)NK(+) severe combined immunodeficiency. *Nat. Genet.* **20**, 394–397 (1998).
18. Fonseca, M. M. *et al.* IL-27 Counteracts Neuropathic Pain Development Through Induction of IL-10. *Front. Immunol.* **10**, 3059 (2019).
19. Fortin, M. *et al.* Spatial and temporal expression of CCR3 and the common beta chain of the IL-3, IL-5 and GM-CSF receptor in the nasal epithelium and lymphoid tissues in a rat model of allergic rhinitis. *Cytokine* **52**, 194–202 (2010).
20. Li, F. *et al.* Protective effect of myokine IL-15 against H2O2-mediated oxidative stress in skeletal muscle cells. *Mol. Biol. Rep.* **41**, 7715–7722 (2014).
21. Yang, J. Y. *et al.* Intestinal Epithelial TBK1 Prevents Differentiation of T-helper 17 Cells and Tumorigenesis in Mice. *Gastroenterology* **159**, 1793–1806 (2020).
22. Fafilek, B. *et al.* Troy, a tumor necrosis factor receptor family member, interacts with lgr5 to inhibit wnt signaling in intestinal stem cells. *Gastroenterology* **144**, 381–391 (2013).

23. Jubb, A. M. *et al.* Achaete-scute like 2 (ascl2) is a target of Wnt signalling and is upregulated in intestinal neoplasia. *Oncogene* **25**, 3445–3457 (2006).
24. Rodriguez-Colman, M. J. *et al.* Interplay between metabolic identities in the intestinal crypt supports stem cell function. *Nature* **543**, 424–427 (2017).
25. Nakanishi, Y. *et al.* Control of Paneth Cell Fate, Intestinal Inflammation, and Tumorigenesis by PKClambda/iota. *Cell Rep.* **16**, 3297–3310 (2016).

Supplementary Fig. 1

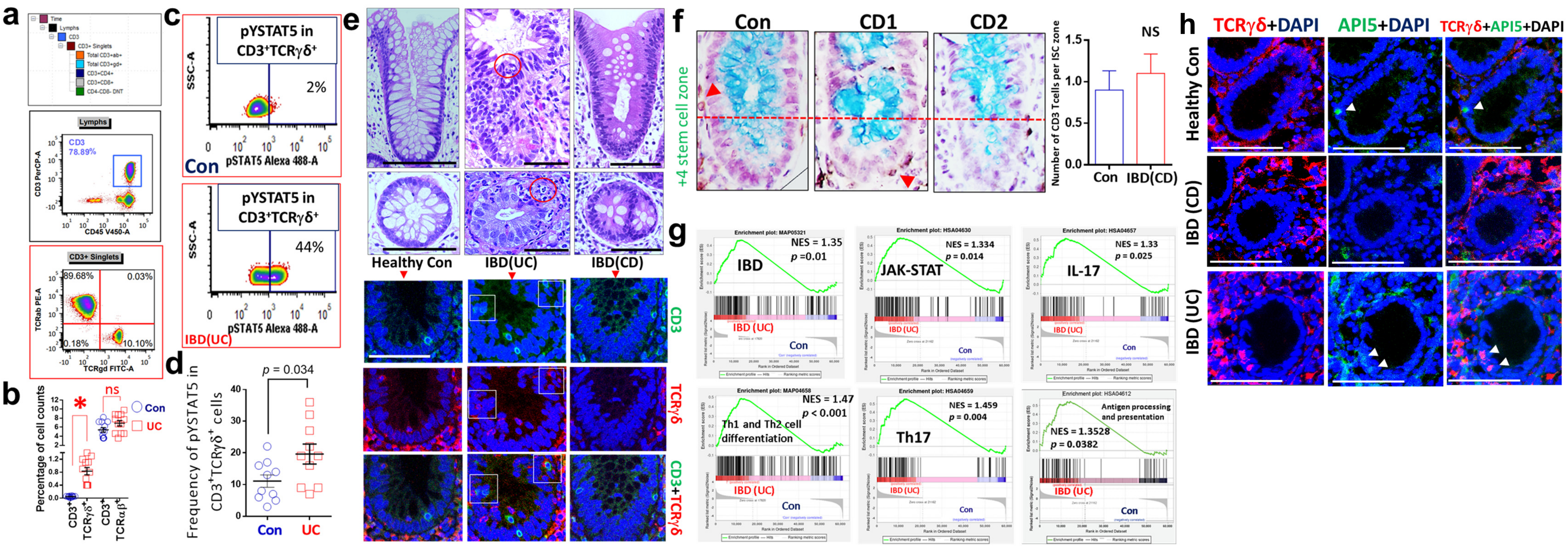

Supplementary Fig. 2

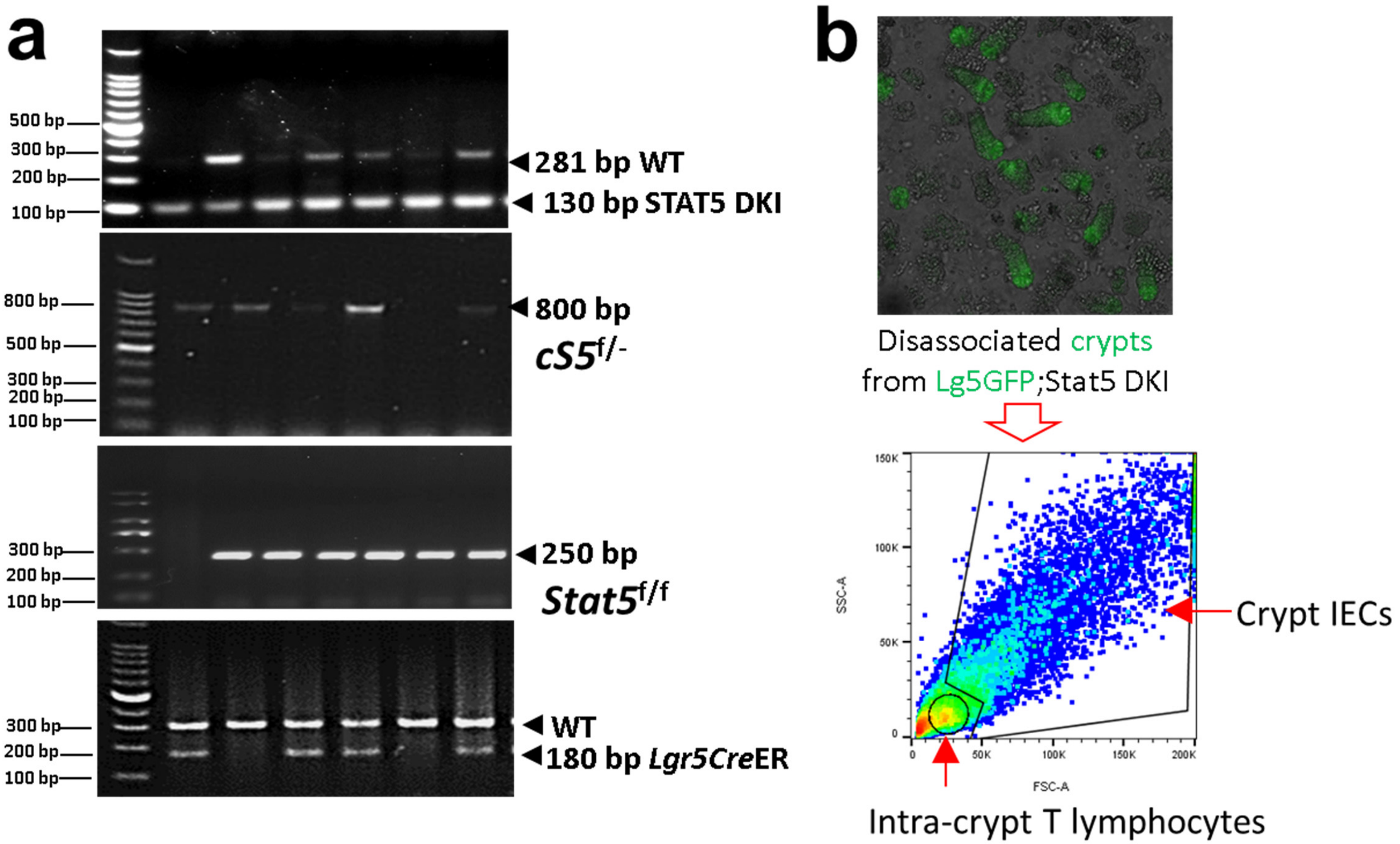

Supplementary Fig. 3

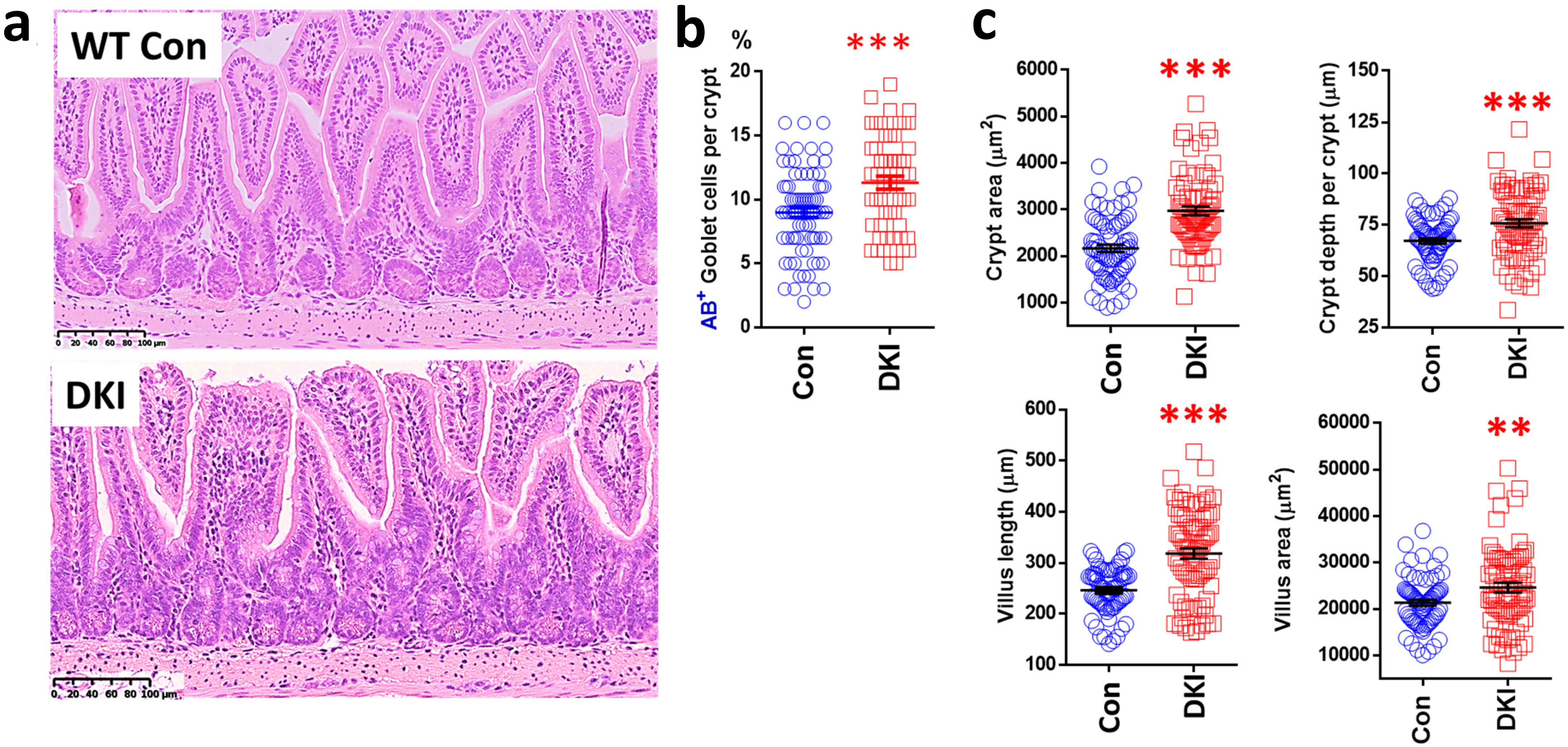

Supplementary Fig. 4

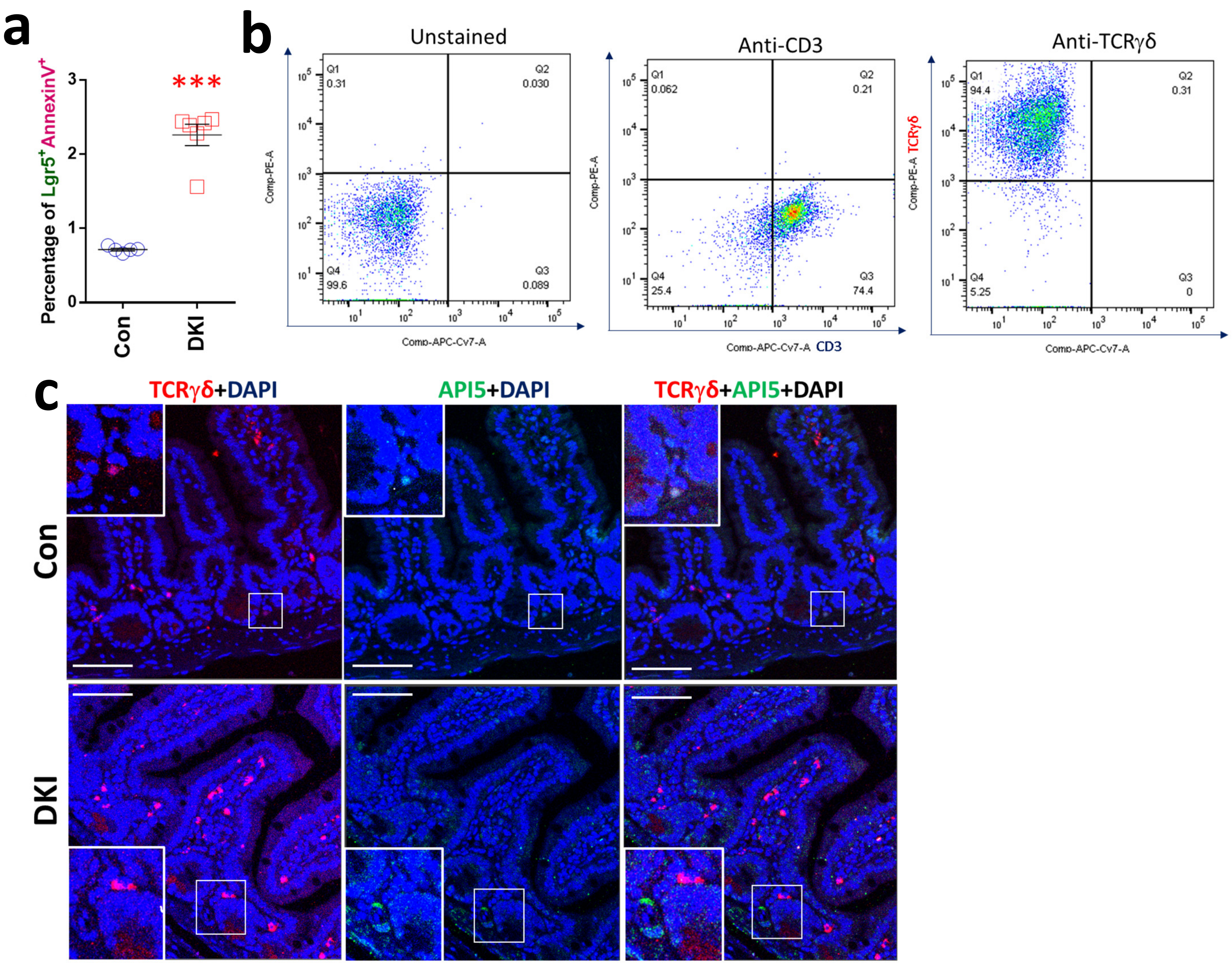

Supplementary Fig. 5

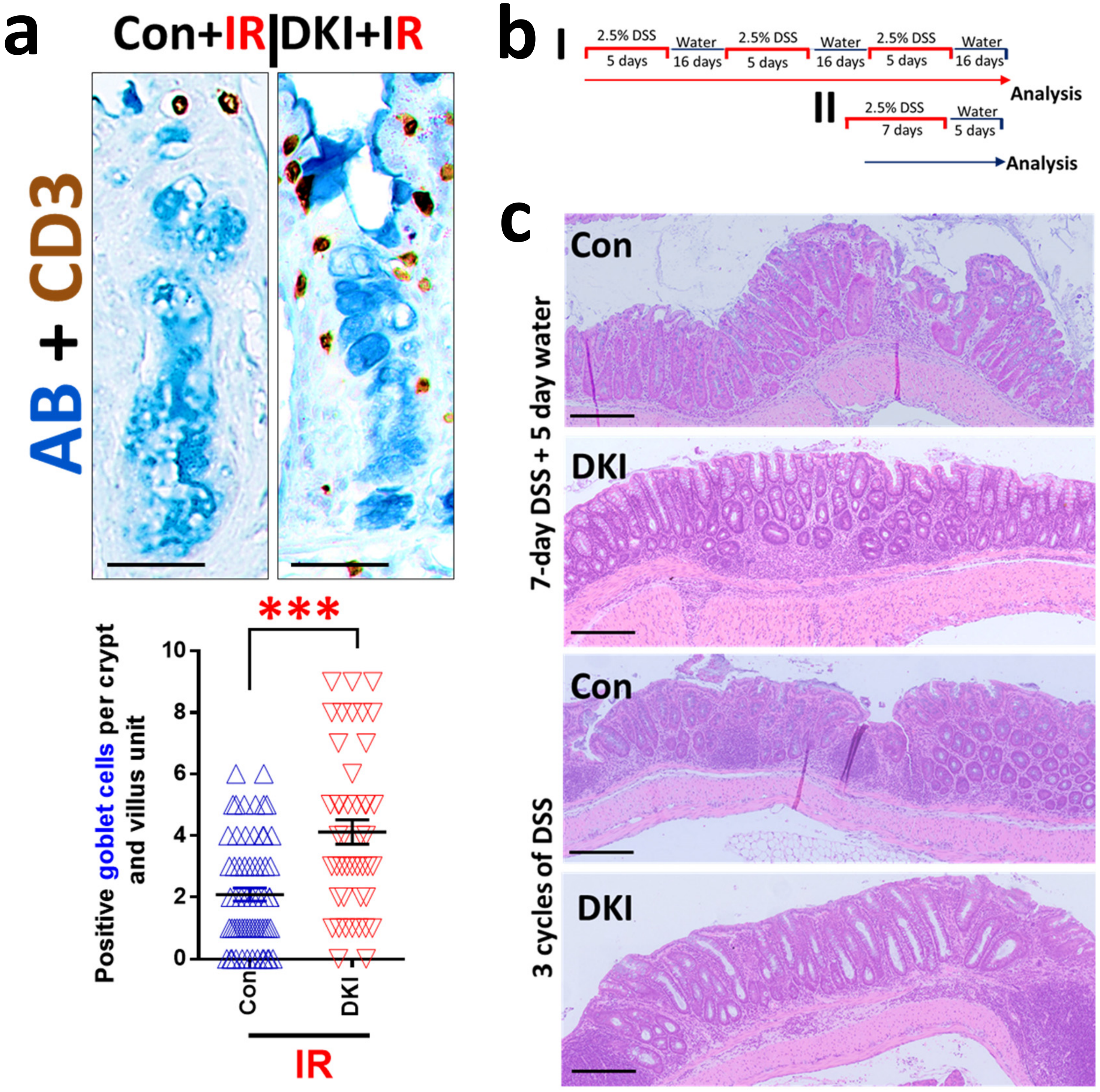

Supplementary Fig. 6

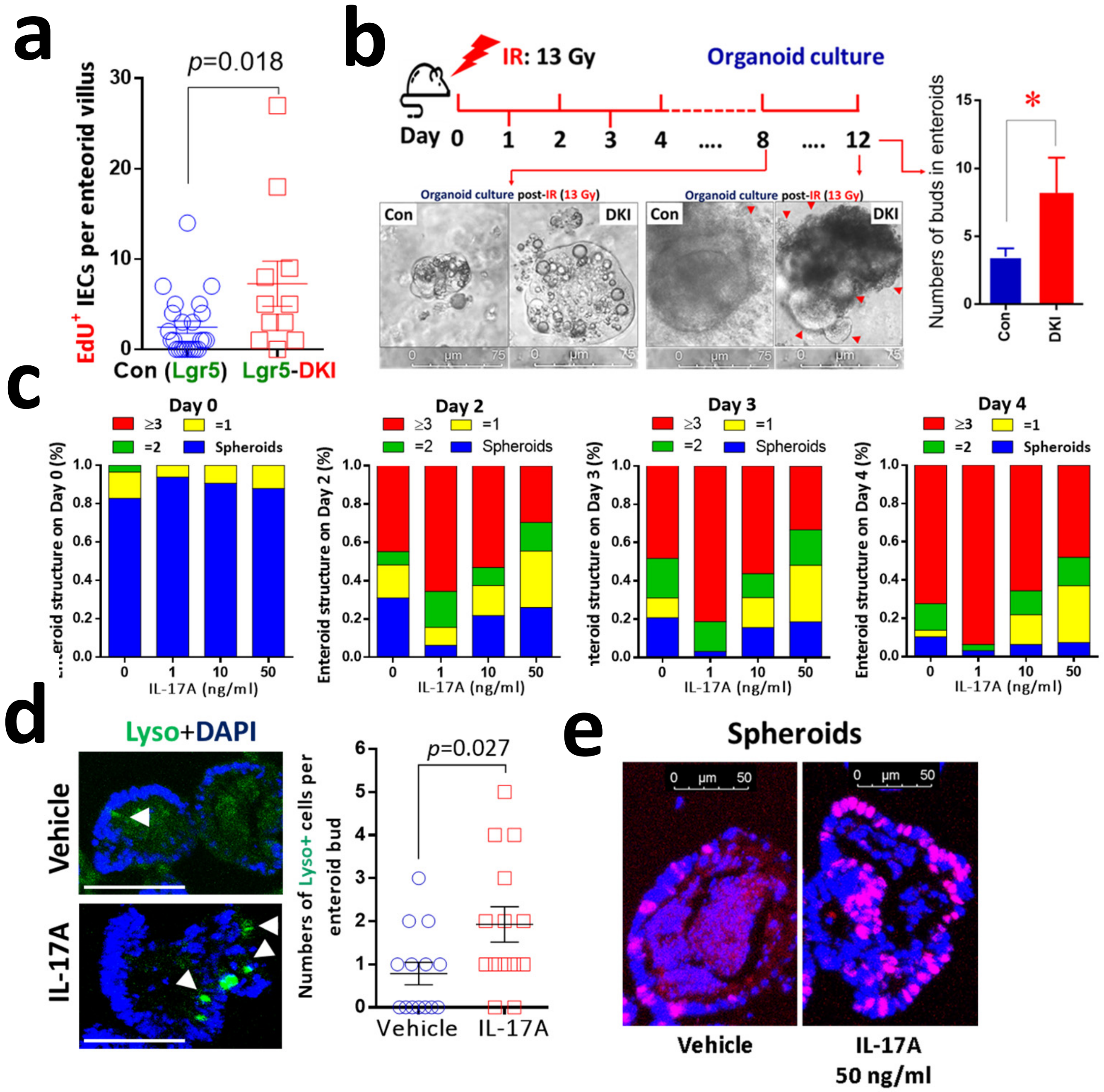

Supplementary Fig. 7

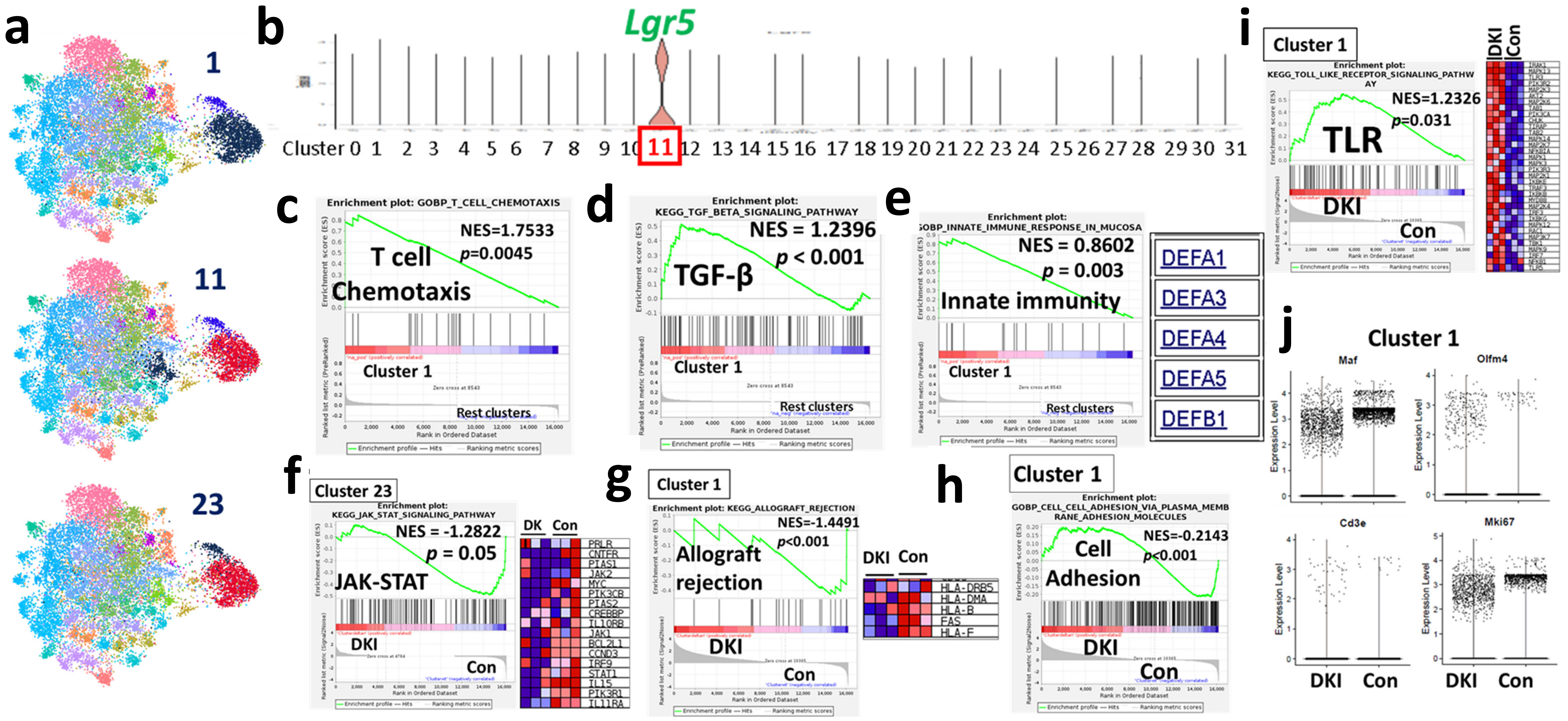

Supplementary Fig. 8

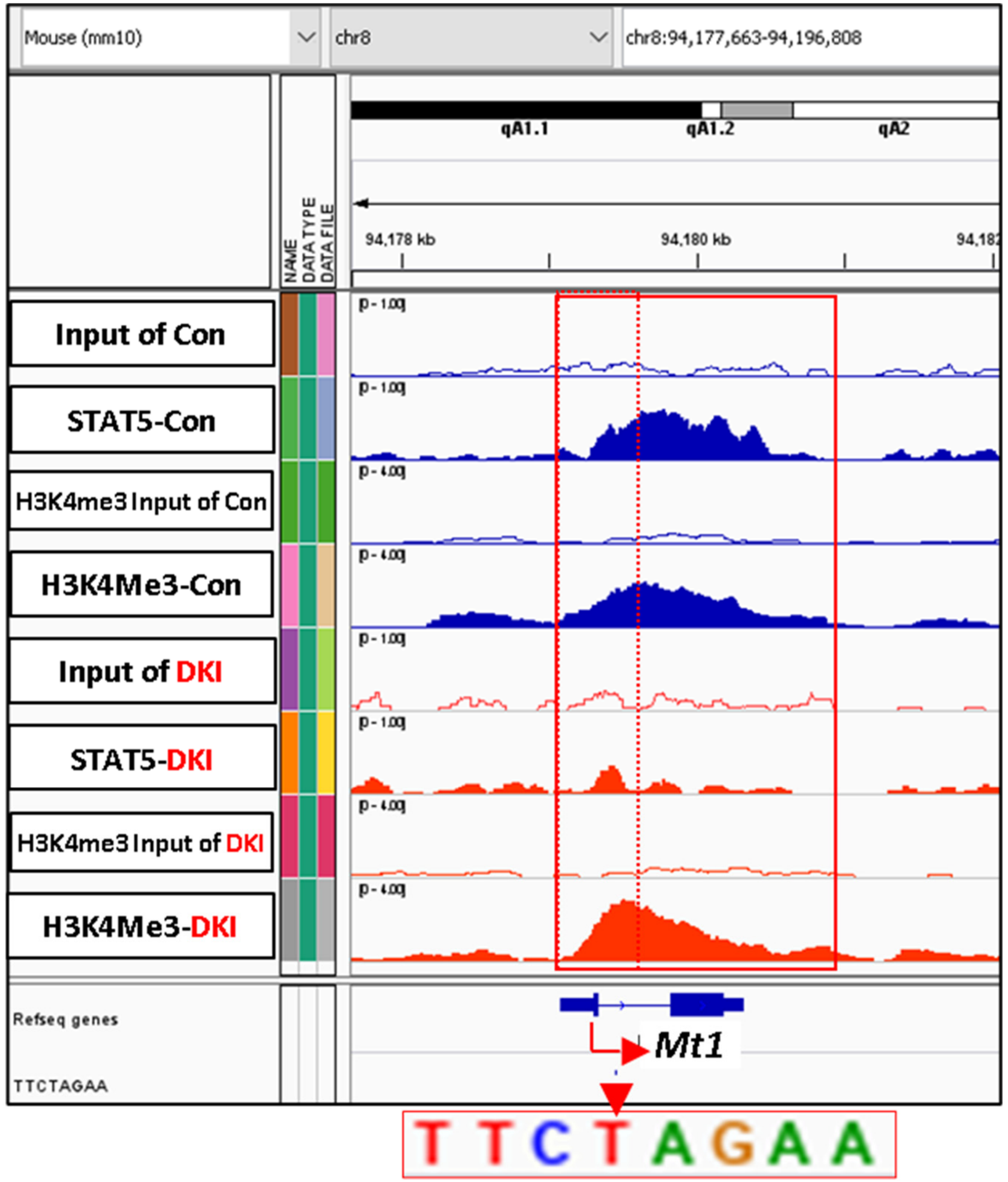

Supplementary Figure Legends

Supplementary Fig. 1. 1×10^6 human PBMCs were obtained from n=10 Con and n=24 UC patients. Pellets were washed with FACS buffer, re-suspended, stained for CD3, CD45, CD4, CD8, TCRαβ, and TCRγδ, and analyzed by FACS. **a** Cell gating to detect $CD3^+TCR\alpha\beta^+$ or $CD3^+TCR\gamma\delta^+$ T cells. Representative FACS gates are shown. **b** The frequency of $CD3^+TCR\alpha\beta^+$ or $TCR\gamma\delta^+$ T cells was determined in the n=10 Con and n=24 UC patients. (**c,d**) pYSTAT5 was determined in the 10 UC patients with elevated $CD3^+TCR\gamma\delta^+$ in blood and 10 healthy control patients. $pYSTAT5^+CD3^+TCR\gamma\delta^+$ T cells were calculated as a percentage of cell counts. The results are expressed as "Mean ± SEM", * $p<0.05$ *vs.* Con. **e** HE staining was performed on 10 Con, 21 CD, and 24 UC patients. Representative images are displayed, and circles indicate intra-IEC lymphocytes. The same crypts were immunostained with anti-CD3 (green) and TCRγδ (red) antibodies. The number of CD3 and TCRγδ double-positive crypt T cells was counted in 50-100 well-orientated colonic crypts as the number of $CD3^+TCR\gamma\delta^+$ crypt T cells per colonic stem cell zone. The results were expressed as "Mean ± SEM". Representative images of CD3- and TCRγδ-double-positive crypt T cells were shown. Bar = 100 μm. **f** The crypt CD3 T cells were stained with CD3 IH in 10 healthy controls and 21 CD patients, and quantified as the number of +4 ISC zone. Intra-crypt $CD3^+$ T cells were counted in 50–100 well-orientated colonic crypts. The results were expressed as "Mean ± SEM". Representative intra-crypt $CD3^+$ T cells are shown in healthy controls and CD patients; "ns" represents "non-significant". **g** RNA was extracted from 3 healthy controls and 7 IBD-UC patients with elevated $TCR\gamma\delta^+$ T cells in PBMC and others, and subjected to RNA-seq analysis. GSEA analysis shows increases in the IBD, JAK-STAT, IL-17, Th17 cell differentiation, and antigen-presenting and -processing pathways in UC *vs.* Con. **h** Apoptosis Inhibitor 5 (API5) and TCRγδ double-IF staining was performed on 5 Con, 9 CD, and 12 UC

patients. Representative images are displayed, and arrows indicate API$^+$ or TCRγδ intra-crypt T lymphocytes, API15 (green) and TCRγδ (red) antibodies. Bar = 100 μm.

Supplementary Fig. 2. a Genotyping of DKI, *Stat5*$^{-/-}$, cS5, and *Lgr5Cre*ER mice. The representative PCR maps are shown. **b** SI crypts were isolated, green Lgr5 crypts and gates of crypt IECs and intra-crypt lymphocyte compartments are shown.

Supplementary Fig. 3. a The SI representative HE images of WT and DKI mice are shown. Bar = 100 μm. **b** The percentage of AB$^+$ goblet cells per crypt was calculated. **c** The alterations of intestinal morphology were measured as crypt area, crypt depth, villus length, and area in DKI *vs.* Con. The results were expressed as "Mean ± SEM". ** p<0.01, *** p<0.001 *vs.* Con

Supplementary Fig. 4. a DKI mice were crossed with *Lgr5Cre*ER mice (Lgr5) to mark LGR5$^+$ ISCs with STAT5 tetramer depletion and sacrificed after EdU i.p. injection. SI crypts from DKI and Con mice were dissociated into single cells and were gated and analyzed by Flow Jo to measure frequencies of 7AAD$^-$Lgr5^{hi}AnnexinV$^+$ crypt IEC cells. **b** The representative scatter graphs of negative staining, CD3$^+$, or TCRγδ$^+$ crypt T cell staining are shown. **c** API5 and TCRγδ double-IF staining was performed on 5 WT Con and 5 DKI mice. Representative images are displayed, and arrows indicate API$^+$ or TCRγδ intra-crypt T lymphocytes, API15 (green) and TCRγδ (red) antibodies. Bar = 100 μm.

Supplementary Fig. 5. a 6 DKI and 5 WT Con mice were sacrificed 3.5 days post-IR. Intestines were isolated, sectioned, and stained by CD3 IH with AB counterstaining. CD3$^+$ crypt IECs were counted, and the result was expressed as "Mean ± SEM"; n≥50 crypts, ** p<0.01, *** p<0.001 *vs.* Con. The bars = 100 μm. **b** The same numbers of DKI and WT Con mice were treated with either 3 cycles of 2.5% DSS with 10-day water recovery in between (I) or 7-day DSS followed by 5-day

water recovery (**II**), n=5 per group. The diagram is shown. **c** The mice were sacrificed. Colonic tissues were isolated, sectioned, and stained with HE. Bar = 200 μm.

Supplementary Fig. 6. a Enteroids from Lgr5-DKI and Lgr5-Con mice were differentiated for 14 days with EdU added on day 10. The EdU$^+$ IECs per enteroid villus were analyzed; >10 enteroids per group were counted. The results are expressed as "Mean ± SEM". **b** DKI and Con mice were treated with 13 Gy IR. At 3.5 days post-IR, the mice were sacrificed. Primary enteroids from the SI crypts of DKI and Con mice were differentiated for 8 days. >10 enteroids per group were traced and counted. The results are expressed as "Mean ± SEM"; * $p<0.05$ *vs.* Con. The media were collected and stored at -80 °C for subsequent medium-transfer experiments. **c** The enteroids were derived from intact Lgr5 crypts dissociated from Lgr5CreER mice. The enteroids were stimulated with IL-17A (1, 10, or 50 ng/ml) for 6 days. Representative GFP or GFP+DIC images of enteroids or IL-17-treated samples were collected on Day 5. The structures of the enteroids were analyzed and expressed as Multiplicity of Enteroids, including spheroids, "1" (single bud), "2" (two buds), and "3" (more than 3 buds). These results are graphed. **d,e** The enteroids treated with 50 ng/ml were incubated with EdU on Day 5, then harvested and fixed with paraformaldehyde (PFA). Sections of 10 μm thickness were stained with anti-Lyz1 (green, **d**) and EdU (pink, **e**). Lyz1+ organoid cells were counted as the number of Lyz1+ cells per organoid. Scatter plots are shown. Results are expressed as the mean ± SEM, n=20 organoids.

Supplementary Fig. 7. SI mucosal cells from 3 DKI and 3 WT Con mice were dissociated into single cells, and 13,000 single cells per sample were analyzed by scRNA-seq. **a** 32 populations of intestinal cells were annotated. Cluster 1 (ISCs + T cells), 11 (stem cells), and 23 (T cells) are shown. **b** Among 32 populations, Lgr5 is highly enriched in cluster 11, indicating that cluster 11 contains a large number of ISCs. **c-e** GSEA analysis shows that compared to the rest of the clusters,

cells in cluster 1 exhibit increased T cell chemotaxis (**c**), activated TGF-β signaling (**d**), and elevated Innate immunity (**e**). NES and p-value are shown. **f-i** Compared to WT Con, GSEA analyses reveal that DKI exhibited significantly reduced JAK-STAT signaling in the T cell population (cluster 23, **f**), significantly reduced allograft rejection pathway along with significantly reduced HLA-DR5, DMA, B and F (**g**), reduced cell adhesion (**h**), and activated TLR pathway (**i**) in the stem cell and T cell population (cluster 1). NES and adjusted *p-values* are shown. **j** DKI exhibits the increased *Maf*, *Cd3e*, *Olfm4,* and *Mki67* genes in cluster 1 compared to cluster 1 in WT Con. Representative scatter graphs are shown.

Supplementary Fig. 8. SI from 3 DKI and 3 Con mice were dissected, and intestinal crypts were dissociated. The nuclear proteins and DNA complex were extracted from 2000 crypts, cross-linked with PFA, and immunoprecipitated with total STAT5 and H3K4Me3 antibodies. Libraries for next-generation sequencing were also prepared and sequenced with a HiSeq 2500 instrument.
